# No Change – No Gain; The Effect of Age, Sex, Selected Genes and Training on Physiological and Performance Adaptations in Cross-Country Skiing

**DOI:** 10.3389/fphys.2020.581339

**Published:** 2020-10-26

**Authors:** Jan-Michael Johansen, Sannija Goleva-Fjellet, Arnstein Sunde, Lars Erik Gjerløw, Lars Arne Skeimo, Baard I. Freberg, Mona Sæbø, Jan Helgerud, Øyvind Støren

**Affiliations:** ^1^Department of Natural Sciences and Environmental Health, University of South-Eastern Norway, Bø, Norway; ^2^Department of Sports, Physical Education and Outdoor Studies, University of South-Eastern Norway, Bø, Norway; ^3^Landslagslegen.no, Top Sports Medical Office, Tønsberg, Norway; ^4^The Norwegian Biathlon Association, Oslo, Norway; ^5^Department of Circulation and Medical Imaging, Norwegian University of Science and Technology, Trondheim, Norway; ^6^Myworkout, Medical Rehabilitation Centre, Trondheim, Norway

**Keywords:** endurance training, skiing performance, training adaptations, double poling, maximal oxygen uptake, lactate threshold, work economy, genomics

## Abstract

The aim was to investigate the effect of training, sex, age and selected genes on physiological and performance variables and adaptations before, and during 6 months of training in well-trained cross-country skiers. National-level cross-country skiers were recruited for a 6 months observational study (pre – post 1 – post 2 test). All participants were tested in an outside double poling time trial (TT_DP_), maximal oxygen uptake in running (RUN-VO_2max_), peak oxygen uptake in double poling (DP-VO_2peak_), lactate threshold (LT) and oxygen cost of double poling (C_DP_), jump height and maximal strength (1RM) in half squat and pull-down. Blood samples were drawn to genetically screen the participants for the *ACTN3* R577X, *ACE* I/D, *PPARGC1A* rs8192678, *PPARG* rs1801282, *PPARA* rs4253778, *ACSL1* rs6552828, and *IL6* rs1474347 polymorphisms. The skiers were instructed to train according to their own training programs and report all training in training diaries based on heart rate measures from May to October. 29 skiers completed all testing and registered their training sufficiently throughout the study period. At pre-test, significant sex and age differences were observed in TT_DP_ (*p* < 0.01), DP-VO_2peak_ (*p* < 0.01), C_DP_ (*p* < 0.05), MAS (*p* < 0.01), LT_v_ (*p* < 0.01), 1RM half squat (*p* < 0.01), and 1RM pull-down (*p* < 0.01). For sex, there was also a significant difference in RUN-VO_2max_ (*p* < 0.01). No major differences were detected in physiological or performance variables based on genotypes. Total training volume ranged from 357.5 to 1056.8 min per week between participants, with a training intensity distribution of 90–5–5% in low-, moderate- and high-intensity training, respectively. Total training volume and ski-specific training increased significantly (*p* < 0.05) throughout the study period for the whole group, while the training intensity distribution was maintained. No physiological or performance variables improved during the 6 months of training for the whole group. No differences were observed in training progression or training adaptation between sexes or age-groups. In conclusion, sex and age affected physiological and performance variables, with only a minor impact from selected genes, at baseline. However, minor to no effect of sex, age, selected genes or the participants training were shown on training adaptations. Increased total training volume did not affect physiological and performance variables.

## Introduction

Cross-country skiing is regarded as one of the most demanding aerobic endurance sports, where male and female athletes have displayed some of the highest maximal oxygen uptakes (VO_2max_) ever recorded (Sandbakk and Holmberg, 2017). VO_2max_, often measured in running (RUN-VO_2max_), is suggested as a main predictor for cross-country skiing and overall endurance performance (Pate and Kriska, 1984; Ingjer, 1991; di Prampero, 2003; Støren et al., 2013; Sandbakk and Holmberg, 2017; Sunde et al., 2019; Johansen et al., 2020). However, in cases where RUN-VO_2max_ is relatively homogenous or held constant, differences in work economy (C) (Conley and Krahenbuhl, 1980; di Prampero, 2003) and/or maximal strength (Hoff et al., 2002; Støren et al., 2008; Sunde et al., 2010, 2019) are regarded as major contributors for differentiating performance in endurance athletes.

Although the main determining factors for cross-country skiing performance are relatively clear, the best way to develop these physiological factors over longer periods in every individual skier is still under investigation (Stöggl and Sperlich, 2015). Traditionally, endurance training makes up almost 90% of the total training for competitive cross-country skiers, while the rest is strength training and speed training (Losnegaard et al., 2013; Stöggl and Sperlich, 2015; Sandbakk et al., 2016). The endurance training during season preparation for both junior and senior cross-country skiers is characterized with high volumes of low-intensity training (LIT) and low to moderate volumes of moderate- (MIT) and high-intensity training (HIT). This has been regarded as an “optimal” intensity distribution for developing higher performance capacity in cross-country skiers (Ingjer, 1992; Seiler and Kjerland, 2006; Sandbakk et al., 2016; Solli et al., 2017). Stöggl and Sperlich (2014) suggests that a polarized training intensity distribution, with high LIT volumes (∼80%) and relatively high HIT volumes (∼20%) with low volumes of MIT, would be more beneficial for further improvements of well-trained endurance athletes, compared to training models with higher volumes of MIT. Additionally, higher volumes of HIT are considered as a more efficient way to elevate VO_2max_ compared to LIT, both in well-trained to elite cross-country skiers and recreational skiers (Nilsson et al., 2004; Helgerud et al., 2007; Støren et al., 2012; Rønnestad et al., 2014, 2016; Stöggl and Sperlich, 2014; Johansen et al., 2020).

Ingjer (1992) observed that young cross-country skiers started to level-off in VO_2max_ at age 19–20 following a training regime similar to that described above, at least in values relative to body mass. Following the same training pattern year after year has not proven to be an effective strategy to increase VO_2max_ further in well-trained and elite adult cross-country skiers (Gaskill et al., 1999; Solli et al., 2017). In Gaskill et al. (1999) and Støren et al. (2012), major changes in the relative intensity distribution of the endurance training led to significant improvements in VO_2max_ and performance in well-trained endurance athletes. However, a recent study showed substantial differences in training response to the same HIT protocol among well-trained cyclists (Bratland-Sanda et al., 2020). This points to the need for better individualization of training programs.

Earlier studies have mainly explored training characteristics in cross-country skiers retrospectively, with no opportunity to investigate the direct physiological effect of the athlete’s training. However, the study of Losnegaard et al. (2013) performed several tests through the preparation phase and the competitive season in elite male cross-country skiers competing at an international and national level. The study revealed improvements in skiing economy (V2 skating), O_2_-deficit and skating performance on a time trial on a roller-skiing treadmill. No improvements were observed in VO_2max_. These were the results of a traditional high volume LIT and low to moderate volume of MIT and HIT regime. However, mainly retrospective studies have been performed on sub-elite and junior cross-country skiers over longer time periods (>10 weeks). No studies have investigated training characteristics and the subsequent physiological effects in both sub-elite senior and junior cross-country skiers competing at a national and regional level over longer periods.

Sex differences in performance determining factors in cross-country skiing is generally reported to be between 10 and 30%, where greater sex differences are shown when the upper-body is used more extensively (Sandbakk et al., 2014; Hegge et al., 2016; Sunde et al., 2019). Sex differences have been examined in recent years among cross-country skiers, however, sex comparisons in training responses to a similar training regimen is not well examined in well-trained cross-country skiers. Previous investigations have revealed no difference in training responses between males and females following the same training program in both sedentary and well-trained individuals (Astorino et al., 2011; Støren et al., 2017; Varley-Campbell et al., 2018), suggesting that this may also be the case for well-trained cross-country skiers. Although both junior and senior skiers have been investigated separately (Ingjer, 1992; Sandbakk et al., 2010, 2016; Losnegaard et al., 2013), direct comparisons of training responses in these age-groups have not been executed previously in cross-country skiers. Investigations of both sex and age-related differences in training responses may be crucial to understand differences in training adaptations, and further improve the quality of the individualization of training programs.

The genetic component of sports performance and trainability has received increasing attention the last two decades. Sports performance is considered a complex trait, influenced by many genes. A number of single nucleotide polymorphisms (SNPs) have been associated with various aspects of athletic ability and sports performance. Two polymorphisms that have been intensely investigated are the *ACTN3* R577X and *ACE* I/D (Jacques et al., 2019). The *ACTN3* gene codes for α-actinin-3, a protein expressed in fast-twitch muscle fibers. The common R577X polymorphism leads to the deficiency of the protein in individuals with the XX genotype (North et al., 1999), which is the case for around 19% of Caucasians (Roth et al., 2008; Goleva-Fjellet et al., 2020). Lack of the α-actinin-3 has been associated with increased muscle endurance, and decreased maximal power generation (MacArthur et al., 2008). The *ACE* gene encodes the angiotensin I-converting enzyme, having a role in the regulation of blood pressure, fluid-electrolyte balance and affecting the muscle function (Puthucheary et al., 2011; Pescatello et al., 2019). *ACE* seems to play a role in exercise induced adaptations and the I allele has been regarded as the endurance allele (Ma et al., 2013; Pescatello et al., 2019). Few studies have investigated these polymorphisms in relation to cross-country skiing performance. Magi et al. (2016) found higher frequencies of the *ACTN3* RR and *ACE* ID genotype in male skiers compared to controls. In addition, male skiers with XX genotype tended to exhibit greater increase in VO_2peak_ over a 5-year period. The same finding applied to female skiers with the ID genotype. Orysiak et al. (2013), on the other hand, did not find any associations between the *ACE* I/D and VO_2max_ in well trained winter sports athletes. No previous studies have compared the genotype distribution for selected genes between regional to national cross-country skiers and the normal population within the same region. Goleva-Fjellet et al. (2020) genotyped *ACE* and *ACTN3* in a cohort representing the region of South East Norway, making it possible to compare this with an athletic cohort.

The *PPARGC1A* rs8192678 SNP has also gained attention in exercise genetics. The protein encoded by the gene, PGC1α (peroxisome proliferator-activated receptor gamma co-activator-1-alpha), induce the mitochondrial biogenesis and modulate the composition and functions of the mitochondria (Austin and St-Pierre, 2012). Recent reviews have concluded that the rs8192678 polymorphism is associated with aerobic trainability and sports performance (Petr et al., 2018, 2020; Tharabenjasin et al., 2019). Peroxisome proliferator-activated receptor genes, e.g., *PPARG* (rs1801282) and *PPARA* (rs4253778), have also been investigated in relation to trainability and athletic ability (Petr et al., 2018, 2020). According to Bouchard et al. (2011) the rs6552828 SNP of the acyl-CoA synthase long-chain member 1 gene (*ACSL1*) could explain around 6% of the training response of VO_2max_ to standardized exercise training programs. A recent study by Harvey et al. (2020) reported that the rs1474347 polymorphisms in the interleukin-6 (*IL6*) gene was associated with training induced improvements in VO_2max_ in both moderately and well trained participants.

To the best of our knowledge, no study have investigated effects of sex, age, training and selected genes on physiological and performance adaptations in the same study. Therefore, the primary aim of this study was to investigate training adaptations in physiological and performance variables in well-trained cross-country skiers after 6 months of training during season preparation (i.e., May to October). Secondly, we wanted to investigate possible differences between gender and age groups in baseline values and training adaptations during the study period. Thirdly, we wanted to investigate the effects of specific candidate genes on physiological and performance variables at baseline. We hypothesized that age and sex would influence on baseline values, but not training adaptations, and that differences in training would impact training adaptations. Further, we hypothesized that the distribution of the selected genetic variants would represent the distribution of the general population for this region and not impact physiological or performance values at baseline.

## Materials and Methods

### Experimental Approach

The main purpose of this study was to evaluate changes in physiological and performance variables after 6 months of training (May to October) in well-trained cross-country skiers. We also wanted to compare baseline values and training induced changes in males and females, and young and older skiers, as well as in skiers with different genotypes. Therefore, the participants were instructed to train according to their own training programs worked out by themselves or their coaches prior to the research project, and report their daily training for the whole 6 months period. They were tested for a number of physiological, strength and performance variables over 2 days at three occasions; before (PRE), mid-way (POST1) and after (POST2) the study period. The test battery consisted of measurements of RUN-VO_2max_, VO_2peak_ in double poling (DP-VO_2peak_), time to exhaustion (TTE), oxygen cost of double poling (C_DP_), lactate threshold in double poling (LT), jump height, 1RM and maximal power tests in half squat and pull-down and performance in a 5.64 km double poling time trial (TT_DP_). At baseline, blood samples were drawn to assess gene status in selected genes.

### Subjects

A total of 46 well-trained cross-country skiers (30 males and 16 females), differing in age (16–48 years) and performance-level, were recruited for the whole study. The study’s medical doctor approved all participants for participation. However, 17 skiers were excluded because they were not able to fulfill the requirements of three testing sessions during the study period due to sickness or injuries or did not report their training habits sufficiently. Thus, 29 skiers were included in the statistical analyzes. To investigate age-related effects the included skiers were divided in two age groups (16–18 and ≥19 years). These groups were defined as either in, or above puberty, and also corresponding to in, or above high-school age. The ≥19 group included skiers from 19 to 48 years. All subjects were recruited by invitation to high-schools for skiers in Southeastern Norway or regional cross country ski teams. The included skiers differed substantially in performance level, from medium-junior level to top national level. The best male and female skiers had finished top 10 in numerous VISMA ski classics races (i.e., Vasaloppet and Marcialonga) and/or top 30 in the Norwegian national championship, and the slowest skiers finished in the lower part of national junior competitions. Subjects‘ characteristics are summarized in Table 1.

**TABLE 1:** Subjects characteristics.

**Variable**	**Total (*n* = 29)**	**Males (*n* = 17)**	**Females (*n* = 12)**	**16–18 years (*n* = 16)**	**≥19 years (*n* = 13)**
Age (yr)	22.1 ± 8.4	24.1 ± 10.2	19.3 ± 4.1	17.3 ± 0.8	28.0 ± 9.8
Weight (kg)	69.4 ± 9.3	73.2 ± 8.6	64.0 ± 7.8**	64.4 ± 6.7	75.5 ± 8.5^§§^
Height (cm)	176.2 ± 8.9	181.1 ± 7.1	169.3 ± 6.3**	173.8 ± 7.7	179.2 ± 9.7
**RUN-VO_2max_**					
mL⋅kg^−1^⋅min^−1^	62.9 ± 8.0	67.4 ± 6.7	56.5 ± 4.5**	61.1 ± 8.0	65.2 ± 7.7
L⋅min^−1^	4.38 ± 0.88	4.92 ± 0.68	3.60 ± 0.37**	3.94 ± 0.70	4.92 ± 0.79^§§^
**Training**					
min⋅week^−1^	241.0 ± 162.6	604.2 ± 153.1	462.1 ± 142.9*	529.4 ± 180.6	557.9 ± 138.7

Values are mean and SD. Yr, years. Kg, kilograms. Cm, centimeters. RUN-VO_2max_, maximal oxygen uptake in running. mL⋅kg^–1^⋅min^–1^, milliliters per kilogram bodyweight per minute. L⋅min^–1^, liters per minute. min^–1^ week, average weekly training the last 3 months in minutes. * *p* < 0.05 significantly different from male value. ** *p* < 0.01 significantly different from male value. §§ *p* < 0.01 significantly different from 16 to 18 years value.

The study was conducted in accordance with the Declaration of Helsinki, and evaluated and approved by the regional ethics committee of Southeast Norway (REK 2017/2522) and the institutional research board at the University of South-Eastern Norway (former University College of South-Eastern Norway). After having received information about the study, all participants gave their written informed consent before participation. Parental written consent was collected for skiers below 18 years.

### Test Procedures

In order to evaluate changes in physiological and performance variables related to the skiers training, all participants were tested at three separate occasions. PRE were performed in April/May, POST1 were performed in July/August, and POST2 were conducted in October/November. All testing procedures were the same at all testing sessions.

All tests were performed on two consecutive days. The participants were instructed to do only light training the last 24 h before testing, and no food or nutritious drinks were allowed 1 h before the first test. In between tests, the participants were allowed to eat a light meal of energy-rich food and drinks. The last meal before testing and food intake in-between tests were registered, and all participants were asked to consume the same food in the subsequent testing sessions (POST1 and POST2). All preparation procedures were the same at all three testing sessions. The tests were also conducted at approximately the same time of day (±2 h) at PRE, POST1 and POST2 to avoid circadian differences.

The first day of testing consisted of three maximal jump height tests, an incremental running test for determining RUN-VO_2max_, and a TT_DP_. Before the jump tests, the participants performed a self-conducted warm-up procedure of at least 10 min. This warm-up was registered and repeated at POST1 and POST2. Then they performed three separate jump tests in the following order: squat jump (SJ), counter-movement jump (CMJ) and counter-movement jump with arm swing (CMJas). For the SJ tests, the knee-angle were 90° and this was controlled by the same test leader at all tests. No counter-movements were allowed in this particular test, whereas no counter-movement restrictions were given for the CMJ and CMJas tests. All participants were given at least three consecutive attempts in each jump-test, and the best attempt was registered as the result. At least 3 min of rest were given between the separate jump tests to ensure sufficient restitution. All jump-tests were performed by use of a force platform (Ergotest Innovation, Porsgrunn, Norway) for jump height measurements. The force platform was calibrated in accordance with the manufacturers’ manual before each test. Jump height was calculated by the following equation,

(1) $$h=\frac{v_{v}^{2}}{2\times g}$$

where *h* is jump height, *v* is the velocity at take-off, which again is based on calculation of force multiplied with time divided by mass, and *g* is gravitation (Ergotest Innovation, Porsgrunn, Norway).

After at least 20 min of rest, the participants started a 10 min self-conducted warm-up procedure before an incremental VO_2max_ test in running. This warm-up was registered and repeated at POST1 and POST2. The RUN-VO_2max_ test was conducted by the same procedures as presented in Sunde et al. (2019). Briefly, the participants started at an intensity of 6% inclination and 7–8 km⋅h^–1^ and 9–10 km⋅h^–1^ for female and male, respectively. The test started with 1% increase in inclination every 30 s until 8% was reached, whereas only speed was increased by 0.5 km⋅h^–1^ every 30 s after that. All participants were instructed to run to voluntary fatigue, and the three highest subsequent VO_2_ measurements were used to calculate VO_2max_. Heart rate (HR) ≥ 98% of HR_max_, respiratory exchange ratio (RER) ≥1.05, blood lactate concentration ([La^–^]_b_) ≥ 8.0 mmol⋅L^–1^, rate of perceived exertion (Borg scale 6–20) ≥17, and flattening of the VO_2_ curve was used to evaluate if VO_2max_ was reached. The metabolic test system, MetaLyzer II Cortex (Biophysic GmbH, Leipzig, Germany) was used for all VO_2_ measurements, with measurements every 10 s. Before testing the O_2_-analyzer were calibrated with ambient air and certified calibration gases (16% O_2_/4% CO_2_), while the flow sensors were calibrated with a 3-L calibration syringe (Biophysic GmbH, Leipzig, Germany) before each test. The treadmill used was a Woodway PPS 55 sport (Waukesha, WI, United States), calibrated for speed and incline. HR were registered by the participants own heart rate monitors or by Polar s610 HR monitors (Kempele, Finland).

After at least 1 h of rest, a 5.64 km TT_DP_ test was performed in a paved roller ski course track of 940 m. The TT procedures have been previously presented in Sunde et al. (2019). Only the DP technique was allowed throughout the test. The TT was organized with individual starts, and 30 s starting intervals. Drafting was not allowed. The subjects used their own roller-skis for classic skiing and poles and were instructed to use wheel type 2 for the time trial test. All subjects used the same pair of roller skis at PRE, POST1, and POST2. Differences in temperature and humidity may influence the rolling resistance of the roller skis, and thus the results of this test. Therefore, we used the same procedures for calculating a correction factor described previously in Sunde et al. (2019).

The second day of testing consisted first of sub-maximal VO_2_ and [La^–^]_b_ measurements in DP, in order to determine C_DP_ and LT. This was, after 5 min of active recovery, followed by a ramp protocol to exhaustion to determine DP-VO_2peak_. After 1 h of rest, the second day of testing ended with two maximal strength tests in half-squat and pull-down.

The DP tests were performed on a motorized treadmill specialized for cross-country skiing (Rodby RL 2700E, Rodby Innovation, Vänge, Sweden). Every participant performed one 30-min workout for familiarization to the DP treadmill before testing, as previously used in Sunde et al. (2019). All participants used the same pair of roller skis at all DP tests during the study period (Swenor Fiberglass, Sarpsborg, Norway) with the same binding system (NNN, Rottefella, Klokkarstua, Norway). The subjects were allowed to use their own poles and additional skiing equipment, which was the same in all three test sessions. During treadmill testing, the participants were attached to a safety harness, connected to the roof, to avoid falling. Three to six 4-min work periods, with registration of VO_2_ and HR measurements the last minute, were conducted for calculating C_DP_ at LT intensity and LT. Work periods were only separated by 1-min for measurements of [La^–^]_b_. Whole blood lactate values were measured by a Lactate Scout+ (SensLab GmbH, Leipzig, ray Inc., Kyoto, Japan). The subjects started the first work period at a work intensity assumed to be 50–70% of their DP-VO_2peak_. This corresponded to 10–11.5 km⋅h^–1^ and 4% inclination for males and 6–8 km⋅h^–1^ and 4% inclination for females. In the following work periods, the speed increased by 1–3 km⋅h^–1^, and the test terminated after [La^–^]_b_ levels exceeding the subjects’ LT. Warm up lactate value (i.e., the lowest measured lactate value) + 2.3 mmol⋅L^–1^ were used to define LT. This is in accordance with the protocol from Helgerud et al. (1990) and described and discussed in detail in Støren et al. (2014) and Sunde et al. (2019).

After 5-min of active rest, the subjects performed the RAMP protocol to exhaustion for determining DP-VO_2peak_. The starting intensity was set to 6% inclination and 7 km⋅h^–1^ for both genders. The inclination was constant through the whole test, while speed increased by 1 km⋅h^–1^ every 60 s. All participants received motivational feedback throughout the test. The test terminated when the skiers slowly moved backward, despite intense motivational feedback, and reached a pre-defined mark 1 m behind the subjects starting position on the treadmill. TTE was registered and the DP-VO_2peak_ was defined as the mean of the two highest subsequent VO_2_-measurements. Maximal aerobic speed (MAS) in double poling were calculated in the same way as presented in Sunde et al. (2019) and Johansen et al. (2020), i.e., DP-VO_2peak_/C_DP_.

A 60-min rest period were given prior to the tests of 1RM and maximal power output in half-squat (Smith-machine, PreCore, Woodinville, WA, United States) and pull-down (Gym 2000, Vikersund, Norway). Pilot testing in Støren et al. (2008) showed no deterioration in 1RM half-squat 30 min after maximal aerobic tests, thus we considered 60-min to be more than sufficient to give valid maximal strength results. The strength tests protocol is identical to the protocol used in Sunde et al. (2019). Both strength tests started with 10 reps at approximately 50% of 1RM. After this, the following sets were performed at approximately 60% (5 reps), 70% (3 reps), and 80% (2 reps), only separated by 3 min rest periods. All repetitions were performed with a slow eccentric phase with a complete stop of movement in the lowest position (half-squat) or the highest position (pull-down) of approximately 1 s. This was followed by a maximal mobilization in the concentric phase. The MuscleLab system (Ergotest Innovation, Porsgrunn, Norway) calculated power output by measurements of lifting time and distance of work. After the sub-maximal series, the participants performed at least 1 rep at their estimated 1RM. From there on: 1 rep, and load increments of 2.5–10 kg from the subsequent lift, were conducted until 1RM was reached.

### Training Registration

The participants were instructed to train according to their own training plans worked out by themselves or by their coaches throughout the study period, without any influence or interventional instructions from the research personnel. All participants recorded training data in digital training diaries, i.e., in an online diary from the Norwegian Olympic Federation, or in Polar Flow. The athletes had all used digital training diaries for at least 1 year prior to the study. Every training session and competition was recorded and controlled by the same research personnel throughout the study period, and 3-months prior to PRE. The two training periods between PRE to POST1 and POST1 to POST2 were defined as 1st training period (*P*_1_) and 2nd training period (*P*_2_). In order to investigate potential changes in training inside *P*_1_ and *P*_2_, the periods have been further divided into a total of four periods where appropriate (*P*_1A_, *P*_1B_, *P*_2A_, and *P*_2B_).

All training data were systemized based on training modality and training intensity. Training modality was either endurance, strength, speed/jump or other, and activity was running, roller-skiing, cross-country skiing or cycling. Roller-skiing and cross-country skiing on snow were defined as ski-specific training, while running and cycling was defined as unspecific training. Endurance training intensity were monitored as HR “time in zone,” and categorized into three intensity zones: (1) low-intensity training (LIT; ≤81% of HRmax), (2) moderate-intensity training (MIT; 82–87% of HRmax), and (3) high-intensity training (HIT; ≥88% of HRmax). All endurance training and competitions were performed with the skiers’ personal heart rate monitors. This is in accordance with the procedures used in Støren et al. (2008) and Sunde et al. (2010).

Strength training consisted mainly of maximal strength training and/or general strength training. Maximal strength training was targeting large muscle groups, i.e., 1–6 repetitions in, i.e., half squat, pull-down or deadlift. General strength training was performed with 10–30 repetitions and with a main purpose of increase stability and general strength in the upper-body and trunk. The duration of strength training sessions where quantified as the time between the first set of the first exercise and last set of the last exercise, including rest periods between sets and exercises. Additional warm-up and cool-down were registered as LIT, while stretching where included in “other training.” Jump training (i.e., 1–6 box-jumps or jump exercises in stairs) was quantified in the same manner as strength training. Speed training during LIT- or MIT-sessions was mainly performed during ski-specific training. The number of sprints were multiplied by 1.5 min since the period after each sprint was performed at a very low intensity. The monitoring of strength-, speed-, and jump training is in accordance with the quantification procedures used in Sandbakk et al. (2016).

### DNA Sampling and Genotyping

Venous blood was drawn when the participants first attended to the laboratory before the physiological testing procedures at the first testing session (April/May). The EDTA tubes were stored at −20°C. Before the DNA extraction, the samples were thawed at room temperature. DNeasy Blood & Tissue Kit (Qiagen, MD, United States) was used to extract the DNA from 100 μl of blood following the manufacturer’s instructions.

*ACE* I/D polymorphism, rs4343 polymorphism in the *ACE* gene was genotyped as it might be the best proxy to I/D polymorphism (Abdollahi et al., 2008), than analyzed to determine the I/D genotype. Genotyping for all polymorphisms was performed using TaqMan^®^ SNP Genotyping Assay. Assay IDs were as follows: C__11942562_20 for *ACE* rs4343; C____590093_1 for *ACTN3* R577X; C__30469648_10 for *ACSL1* rs6552828; C___1643192_20 for the *PPARGC1A* rs8192678; C___1839698_20 for *IL6* rs1474347; C___1129864_10 for *PPARG* rs1801282 and C___2985251_20 for *PPARA* rs4253778 polymorphism (Thermo Fisher Scientific, MA, United States). StepOnePlus^TM^ Real-Time PCR System (Applied Biosystems^®^, CA, United States) was used to carry out the qPCR. Genotype calling was performed by StepOne Software v2.0. 15 μl of final reaction volume contained 8.44 μl Genotyping Master Mix, 0.42 μl Assay mix (40×), 6.33 μl double distilled H_2_O and ∼100 ng of DNA template. Cycling conditions were as follows: 30 s at 60°C was followed by initial denaturation step for 10 min at 95°C; then, 40 cycles of denaturation at 95°C for 15 s were followed by annealing at 60°C for 1 min in cycling stage, finishing with the final post-read step for 30 s at 60°C.

### Statistical Analyzes

Normality tests and Q-Q plots were used to evaluate normal distribution for main variables (TT_DP_, RUN-VO_2max_ and MAS). In all cases, a normal distribution was observed, thus parametric statistics were used. Values were expressed as mean ± SD, and inter-individual variability in training and physiological variables were expressed as coefficient of variance (CV). To evaluate potential changes in physiological response and training characteristics for the total group, within sexes and within age groups, a Univariate General Linear Model (GLM) test with Tukey *Post Hoc*-tests was used. To examine potential differences between sexes and age groups in physiological response and training characteristics during the study period, GLM Univariate with pairwise comparisons and independent sample *t*-tests were conducted. For correlations between baseline values, and between differences between different test points (delta correlations), correlation coefficients *r* was used from Pearson’s bivariate tests. Correlation coefficients were evaluated in accordance with Hopkins (2000), which are presented in detail previously (Sunde et al., 2019). Since the participants represented both female and male skiers, also partial correlations were conducted corrected for sex and age.

One-way ANOVA with Tukey *Post Hoc*-tests was used to assess the associations between the genotypes and physiological and performance variables at baseline. To assess the effects of the alleles on these variables, a two-tailed independent sample *t*-test was applied. In order to test for the Hardy-Weinberg equilibrium (HWE) for all polymorphisms and to compare the genotype frequencies to those of other studies, Pearson’s Chi-square test (*χ*^2^) was used. When analyzing effects of different genotypes on physiological parameters, all female values from the physiological tests were multiplied according to the average gender difference between males and females in the present study. This was conducted to avoid bias effects of different gender representation for the different candidate genes and genotypes. In order to promote comparability between candidate gene studies, effect size (Cohen’s *d*) was calculated using Microsoft^®^ Excel^®^ (Redmond, WA, United States) for the gender corrected variables across the genotypes (Supplementary Table 6). The effect size was interpreted as follows: below 0.50 – small effect, 0.5 and above – moderate effect, 0.8 and above – large effect (Cohen, 1988). As the participants were following individual training programs, genetic analyzes of trainability were not performed. For all statistical analyzes performed, the statistical package for social science version 26 (SPSS, IBM, Chicago, IL, United States) was used. A *p* value < 0.05 was accepted as statistically significant in all tests (two-tailed).

Power calculations prior to the study revealed that with a between-group difference in the selected physiological variables of 5%, and with a common standard deviation of the same size, a sample size of 12 to 16 subjects were needed in each age- and gender group in order accomplish a significant level of 0.05 and a power of 80%. Regarding the genetic variables, the material is under-powered in order to accomplish full genetic analyses. Multivariate ANOVA analyzes between the different genotypes and the different physiological variables were thus not performed. However, the material was still interesting in order to see if there were substantial differences in physiological variables related to single genes. Also, the material was sufficient to investigate if the cohort of skiers differentiated from a general population from the same geographical area in genotype and allele frequencies.

## Results

### Training Characteristics

The skiers training was registered for 23.4 ± 2.2 weeks from PRE to POST2. From PRE to POST1 the skiers trained for 12.7 ± 1.7 weeks, and for 10.7 ± 1.4 weeks from POST1 to POST2. In total, 8460 training sessions were registered, with 5957 inside the 6-months study period. The remaining sessions registered were conducted in the 3 months before PRE. This corresponded to an average of 205 ± 48 sessions per skier during the study period, and 292 ± 72 sessions per skier when the training period before PRE were included.

Training characteristics for the whole group in *P*_1_ and *P*_2_ are presented in Table 2, while the sub-periods (*P*_1A_, *P*_1B_, *P*_2A_, and *P*_2B_) are presented in Supplementary Table 3. The mean total training volume in *P*_1_ was 701.5 ± 169.8 min⋅week^–1^ and increased significantly to 753.2 ± 137.6 min⋅week^–1^ in *P*_2_ (*p* < 0.05). Total endurance training accounted for 86.9 ± 6.6 and 84.4 ± 7.1% of total training volume in *P*_1_ and *P*_2_, respectively. The relative intensity distribution in the endurance training was 90.0 ± 4.3, 4.8 ± 2.2, and 5.2 ± 3.0% in LIT, MIT, and HIT, respectively, in *P*_1_. In *P*_2_, LIT, MIT, and HIT represented 89.6 ± 3.2, 4.8 ± 2.2, and 5.7 ± 2.4%, respectively. The relative intensity distribution did not change significantly throughout the 6-months training period. Ski-specific training accounted for 49.7 ± 13.6 and 55.7 ± 10.5% of total endurance training in *P*_1_ and *P*_2_, respectively. Total ski-specific training and ski-specific LIT increased significantly from *P*_1_ to *P*_2_ (*p* < 0.01), while ski-specific MIT and HIT remained unchanged. In total, 65.2 ± 18.0 and 62.4 ± 17.7% of ski-specific training was performed as classic skiing, while the remaining 34.8 ± 17.3 and 37.6 ± 17.7% was performed as freestyle-skiing in *P*_1_ and *P*_2_, respectively. Most of the remaining volume of total endurance training were performed either as running (40.1 ± 9.8% in *P*_1_, 38.6 ± 9.0% in *P*_2_) or as cycling (9.9 ± 14.7% in *P*_1_, 5.6 ± 7.0% in *P*_2_).

**TABLE 2:** Training characteristics during the 6 months study period (*n* = 29).

**Variable**	***P*_1_ (May to July)**	***P*_2_ (August to October)**
Duration (weeks)	12.7 ± 1.7	10.7 ± 1.4
**Training (min⋅week**^–^**^1^)**		
Total training volume	701.5 ± 169.8	753.2 ± 137.6*
**Endurance training**		
LIT	548.7 ± 148.2	569.1 ± 116.9
MIT	29.4 ± 11.4	30.4 ± 14.7
HIT	31.8 ± 15.7	36.0 ± 17.2
Total	609.8 ± 154.1	635.5 ± 126.3
**Training mode**		
Ski specific	303.1 ± 120.1	353.8 ± 105.4**
LIT_ski_	270.2 ± 108.0	313.6 ± 91.0**
MIT_ski_	15.7 ± 8.4	19.4 ± 12.4
HIT_ski_	14.0 ± 11.7	17.5 ± 10.6
Running	244.5 ± 77.6	245.4 ± 71.8
Cycling	60.5 ± 95.5	35.346.2
Strength training	61.7 ± 30.5	77.8 ± 31.4**
Speed/jump training	8.2 ± 8.4	9.6 ± 10.4
Other	21. ± 741.8	30.5 ± 33.9

Values are mean and SD with coefficient of variance in percentage. min⋅week^–1^, minutes per week. *P*_1_, first training period from May to July. *P*_2_, second training period from August to October. LIT, low-intensity training. MIT, moderate-intensity training, HIT, high-intensity training. * *p* < 0.05 significantly different from *P*_1_ value. ** *p* < 0.01 significantly different from *P*_1_ value.

Strength training was performed regularly with 1–3 sessions per week throughout the study period. In *P*_1_, strength training accounted for 8.8 ± 4.0% of the total training volume while in *P*_2_, 10.3 ± 3.8% of total training volume was strength training. The amount of strength training increased significantly from *P*_1_ to *P*_2_ (*p* < 0.01). Speed/jump and other training stayed unchanged throughout the whole training period while accounting for 1.2 ± 1.3 and 3.1 ± 4.8% in *P*_1_ and 1.3 ± 1.4 and 4.0 ± 4.1% in *P*_2_, respectively.

### Physiological Adaptations

Results in physiological and performance variables at the three testing sessions (PRE, POST1, and POST2) are presented in Table 3. No significant changes were observed in physiological and performance variables in the whole group from PRE to POST1, from POST1 to POST2, except for RER_*RUN*_ (*p* < 0.05), or PRE to POST2.

**TABLE 3:** Physiological and performance characteristics during the study period (*n* = 29).

**Variable**	**PRE**		**POST1**		**POST2^a^**	
BW (kg)	69.4 ± 9.3	(13.4)	69.0 ± 8.6	(12.5)	69.6 ± 8.3	(11.9)
**TT_DP_**						
seconds	875.1 ± 92.8	(10.6)	866.9 ± 91.4	(10.5)	845.9 ± 88.2	(10.4)
**RUN-VO_2max_**						
mL⋅kg^–1^⋅min^–1^	62.9 ± 8.0	(12.7)	64.7 ± 7.7	(11.9)	64.1 ± 8.8	(13.7)
L⋅min^–1^	4.38 ± 0.87	(19.9)	4.48 ± 0.85	(19.0)	4.47 ± 0.86	(19.2)
mL⋅kg^–0.67^⋅min^–1^	254.6 ± 36.1	(14.2)	261.4 ± 35.4	(13.5)	259.7 ± 38.8	(14.9)
HR	196.6 ± 10.6	(5.3)	195.5 ± 10.6	(5.4)	193.6 ± 10.9	(5.6)
RER	1.12 ± 0.03	(2.7)	1.11 ± 0.05	(4.5)	1.14 ± 0.04*	(3.5)
[La^–^_b_]	10.1 ± 2.3	(22.8)	11.3 ± 2.6	(23.0)	10.0 ± 2.1	(21.0)
RPE	17.2 ± 1.7	(9.9)	17.9 ± 1.2	(6.7)	17.6 ± 1.4	(7.9)
**DP-VO_2peak_**						
mL⋅kg^–1^⋅min^–1^	54.3 ± 7.3	(13.4)	54.6 ± 7.2	(13.2)	55.5 ± 7.3	(13.2)
L⋅min^–1^	3.79 ± 0.79	(20.8)	3.80 ± 0.73	(19.2)	3.89 ± 0.74	(19.0)
mL⋅kg^–0.67^⋅min^–1^	220.0 ± 33.0	(15.0)	221.0 ± 31.7	(14.3)	225.2 ± 32.3	(14.3)
%RUN-VO_2max_	86.5 ± 7.3	(8.4)	84.4 ± 5.8	(6.9)	86.9 ± 5.7	(6.6)
HR	190.8 ± 9.8	(5.1)	190.9 ± 9.9	(5.2)	190.8 ± 9.9	(5.2)
RER	1.10 ± 0.06	(5.4)	1.11 ± 0.05	(4.5)	1.13 ± 0.05	(4.4)
[La^–^_b_]	9.2 ± 2.0	(21.8)	9.0 ± 1.9	(21.1)	9.0 ± 1.7	(18.9)
RPE	17.5 ± 1.2	(6.9)	17.6 ± 1.1	(6.3)	17.5 ± 1.4	(8.0)
TTE (s)	494.3 ± 125.4	(25.4)	524.0 ± 127.9	(24.4)	542.9 ± 124.0	(22.8)
**C_DP_ at LT**						
mL⋅kg^–1^⋅m^–1^	0.198 ± 0.021	(10.6)	0.193 ± 0.019	(9.8)	0.193 ± 0.020	(10.4)
mL⋅kg^–0.67^⋅m^–1^	0.800 ± 0.078	(9.8)	0.779 ± 0.070	(9.0)	0.780 ± 0.066	(8.5)
**MAS**						
m⋅min^–1^	278.1 ± 52.6	(18.9)	285.4 ± 45.1	(15.8)	290.3 ± 44.2	(15.2)
km⋅h^–1^	16.7 ± 3.2	(19.2)	17.1 ± 2.7	(15.8)	17.4 ± 2.7	(15.5)
**LT**						
%DP-VO_2peak_	82.3 ± 6.5	(7.9)	82.4 ± 6.3	(7.6)	81.6 ± 5.6	(6.9)
HR	175.4 ± 11.5	(6.6)	173.2 ± 11.9	(6.9)	172.3 ± 11.9	(6.9)
VO_2_	44.6 ± 6.6	(14.8)	44.9 ± 6.4	(14.3)	45.3 ± 6.8	(3.9)
[La^–^_b_]	4.6 ± 0.6	(13.0)	4.7 ± 0.7	(14.9)	4.5 ± 0.6	(13.3)
Speed (km⋅h^–1^)	13.7 ± 2.5	(18.7)	14.1 ± 2.2	(15.6)	14.2 ± 2.1	(14.8)
**Strength**						
1RM half squat (kg)	120.8 ± 21.9	(18.1)	129.7 ± 24.2	(18.7)	131.1 ± 23.3	(17.8)
1RM pull-down (kg)	87.4 ± 16.5	(18.9)	87.9 ± 15.6	(17.7)	89.8 ± 16.2	(18.0)
**Maximal power**						
Half squat (w)	808.6 ± 207.6	(25.7)	816.6 ± 177.4	(21.7)	831.8 ± 180.7	(21.7)
Pull-down (w)	473.9 ± 152.8	(32.2)	469.7 ± 119.1	(25.4)	490.3 ± 124.0	(25.3)
SJ (cm)	28.0 ± 5.1	(18.2)	26.8 ± 4.7	(17.5)	27.2 ± 4.8	(17.6)
CMJ (cm)	31.5 ± 5.5	(17.5)	31.6 ± 4.2	(13.3)	30.7 ± 5.0	(16.3)
CMJas (cm)	35.9 ± 5.5	(15.3)	35.0 ± 4.9	(14.0)	33.6 ± 5.2	(15.5)

Values are mean and SD with coefficient of variance in percentage in parenthesis. ^*a*^no effect size for delta physiological variables from PRE to POST2 over 0.5. BW, body-weight. Kg, kilograms. TT_*DP*_, double poling time trial. RUN-VO_2max_, maximal oxygen uptake in running. mL⋅kg^–1^⋅min^–1^, milliliters per kilogram bodyweight per minute. L⋅min^–1^, liters per minute. mL⋅kg^–0.67^⋅min^–1^, milliliters per kilogram raised to the power of −0.67 per minute. HR, heart rate. RER, respiratory exchange ratio. [La^–^_*b*_], blood lactate concentration. RPE, rate of perceived exertion. %RUN-VO_2max_, fractional utilization of RUN-VO_2max_ at DP-VO_2peak_. TTE, time to exhaustion. C_*DP*_, oxygen cost of double poling at lactate threshold. mL⋅kg^–1^⋅m^–1^, milliliters per kilogram per meter. mL⋅kg^–0.67^⋅m^–1^, milliliters per kilogram raised to the power of −0.67 per meter. MAS, maximal aerobic speed. LT, lactate threshold. VO_2_, oxygen uptake. Km, kilometers. H, hours. 1RM, one repetition maximum. W, watt. SJ, squat jump. CMJ, counter movement jump. CMJas, counter movement jump with armswing. Cm, centimeters. * *p* < 0.05 significantly different from POST1 value.

Correlations between physiological and performance variables at baseline and between delta values in physiological, performance and training variables is presented in Tables 4–6. Strong correlations were observed between TT_DP_ and DP-VO_2peak_ (*r* = −0.79, *p* < 0.01), MAS (*r* = −0.79, *p* < 0.01), LT_v_ (*r* = −0.82, *p* < 0.01), RUN-VO_2max_ (*r* = −0.68, *p* < 0.01), and 1RM pull-down (*r* = −0.64, *p* < 0.01) at baseline for the whole group. Corrected for gender, strong significant correlations were still apparent between TT_DP_ and DP-VO_2peak_ (*r* = −0.63, *p* < 0.01), MAS (*r* = −0.58, *p* < 0.01), and LT_v_ (*r* = −0.64, *p* < 0.01) at baseline. Corrected for age-groups, the similar strong correlations as seen for the whole group were almost at same level between TT_DP_ and RUN-VO_2max_ (*r* = −0.68, *p* < 0.01), LT_v_ (*r* = −0.77, *p* < 0.01), DP-VO_2peak_ (*r* = −0.76, *p* < 0.01), MAS (*r* = −0.75, *p* < 0.01), and 1RM pull-down (*r* = −0.52, *p* < 0.01). A strong correlation was also apparent between MAS and LT_v_, both independent (*r* = 0.93, *p* < 0.01) and dependent (*r* = 0.85, *p* < 0.01 and *r* = 0.89, *p* < 0.01) of gender and age, respectively.

**TABLE 4:** Baseline correlations between performance and physiological variables (*n* = 29).

**Variable**	**TT_DP_**	**VO_2max_ (mL**^–^⋅**^1^ kg**^–^**^0.67^⋅min**^–^**^1^)**	**MAS**
Age	−0.09(0.23)	0.15 (0.25)	0.07(−0.28)
BW	−0.41*(−0.01)	0.43*(−0.02)	0.40*(−0.03)
**TT_DP_**			
seconds	−	−0.73**(−0.39)	−0.79**(−0.58**)
**RUN-VO_2max_**			
mL⋅kg^–1^⋅min^–1^	−0.68**(−0.33)	0.96**(0.92**)	0.69**(0.34)
L⋅min^–1^	−0.71**(−0.35)	0.92**(0.77**)	0.72**(0.27)
mL⋅kg^–0.67^⋅min^–1^	−0.73**(−0.39)	−	0.75**(0.37)
HR	−0.07(−0.07)	−0.02(−0.003)	−0.03(−0.01)
RER	−0.14(−0.001)	−0.01(−0.34)	0.08(−0.15)
[La^–^_b_]	0.26 (0.24)	0.08 (0.27)	−0.22(−0.23)
RPE	−0.36(−0.45*)	0.06 (0.03)	0.16 (0.21)
**DP-VO_2peak_**			
mL⋅kg^–1^⋅min^–1^	−0.79**(−0.63**)	0.75**(0.44*)	0.81**(0.65**)
L⋅min^–1^	−0.78**(−0.57**)	0.77**(0.34)	0.79**(0.48*)
mL⋅kg^–0.67^⋅min^–1^	−0.83**(−0.69**)	0.80**(0.46*)	0.85**(0.68**)
%RUN-VO_2max_	−0.24(−0.32)	−0.20(−0.46*)	0.24 (0.32)
HR	−0.06(0.08)	−0.001(−0.12)	0.09 (0.03)
RER	0.18(0.49*)	0.11(−0.09)	−0.03(−0.29)
[La^–^_b_]	0.08 (0.21)	0.09(−0.10)	0.11(−0.04)
RPE	−0.01(−0.16)	−0.03(0.14)	−0.04(0.13)
TTE (s)	−0.84**(−0.72**)	0.78**(0.43*)	0.93**(0.85**)
**C_DP_ at LT**			
mL⋅kg^–1^⋅m^–1^	0.41*(0.17)	−0.40*(−0.07)	−0.69**(−0.63**)
mL⋅kg^–0.67^⋅m^–1^	0.27 (0.17)	−0.23(−0.08)	−0.56**(−0.62**)
**MAS**			
m⋅min^–1^	−0.79**(−0.58**)	0.75**(0.37)	−
km⋅h^–1^	−0.79**(−0.58**)	0.75**(0.37)	−
**LT**			
%DP-VO_2peak_	−0.05(−0.13)	−0.03(0.17)	−0.24(−0.19)
HR	0.08 (0.01)	−0.25(−0.19)	−0.22(−0.11)
VO_2_	−0.72**(−0.56**)	0.66**(0.46*)	0.59**(0.37)
[La^–^_b_]	0.25(0.51**)	−0.06(−0.26)	−0.23(−0.51**)
Speed (km⋅h^–1^)	−0.82**(−0.64**)	0.77**(0.48*)	0.91**(0.84**)
**Strength**			
1RM half squat	−0.49**(−0.15)	0.59**(0.33)	0.55**(0.29)
1RM pull-down	−0.64**(−0.27)	0.61**(0.07)	0.60**(0.13)
Power half squat	−0.48**(−0.12)	0.54**(0.10)	0.49**(0.05)
Power pull-down	−0.53**(−0.27)	0.50**(−0.14)	0.54**(0.07)

Values are correlation coefficients *r* and partial correlation coefficients corrected for sex in parenthesis. BW, body-weight. Kg, kilograms. TT_*DP*_, double poling time trial. RUN-VO_2max_, maximal oxygen uptake in running. mL⋅ < *kg* ⋅min^–1^, milliliters per kilogram bodyweight per minute. L⋅min^–1^, liters per minute. mL⋅kg^–0.67^⋅min^–1^, milliliters per kilogram raised to the power of −0.67 per minute. HR, heart rate. RER, respiratory exchange ratio. [La^–^_*b*_], blood lactate concentration. RPE, rate of perceived exertion. %RUN-VO_2max_, fractional utilization of RUN-VO_2max_ at DP-VO_2peak_. TTE, time to exhaustion. C_*DP*_, oxygen cost of double poling at lactate threshold. mL⋅kg^–1^⋅m^–1^, milliliters per kilogram per meter. mL⋅kg^–0.67^⋅m^–1^, milliliters per kilogram raised to the power of −0.67 per meter. MAS, maximal aerobic speed. LT, lactate threshold. VO_2_, oxygen uptake. Km, kilometers. H, hours. 1RM, one repetition maximum. * *p* < 0.05 significant correlation. ** *p* < 0.01 significant correlation.

**TABLE 5:** Delta correlations between physiological and performance variables and training (*n* = 29).

**Variables**	**RUN-VO_2max_**	**DP-VO_2peak_**	**C_DP_**	**MAS**	**LT**_v_	**TT_DP_**
Total training	0.11	−0.01	−0.25	0.19	0.35	−0.13
Ski specific training	0.08	−0.03	−0.34	0.28	0.48**	−0.06
LIT_ski_	0.07	−0.11	−0.41*	0.29	0.45*	−0.05
Strength training	0.15	−0.08	−0.30	0.16	0.36	−0.32

Values are correlation coefficient *r* for delta physiological values from POST1 to POST2, and delta training values that showed significant differences from 1st training period to 2nd training period. RUN-VO_2max_, maximal oxygen uptake in running in milliliters per kilogram body weight raised to the power of −0.67 per minute. DP-VO_2peak_, peak oxygen uptake in double poling in milliliters per kilogram body weight raised to the power of −0.67 per minute. C_*DP*_, oxygen cost of double poling in milliliters per kilogram bodyweight per meter. MAS, maximal aerobic speed. LT_*v*_, velocity at lactate threshold. TT_*DP*_, double poling time trial. LIT_*ski*_, ski-specific low intensity training. * *p* < 0.05 significant correlation. ** *p* < 0.01 significant correlation.

**TABLE 6:** Delta correlations between performance and physiological variables (*n* = 29).

**Variable**	**ΔTT_DP_**	**ΔRUN-VO_2max_**	**ΔMAS**
	**Δ PRE–POST2**	**Δ PRE–POST2**	**Δ PRE–POST2**
ΔBW	0.34	−0.24	−0.31
**ΔTT_DP_**			
seconds	−	−0.05	−0.09
**ΔRUN-VO_2max_**			
mL⋅kg^–1^⋅min^–1^	−0.11	0.99**	−0.21
L⋅min^–1^	0.08	0.93**	−0.20
mL⋅kg^–0.67^⋅min^–1^	−0.05	−	−0.21
HR	−0.11	0.19	−0.39*
**ΔDP-VO_2peak_**			
mL⋅kg^–1^⋅min^–1^	−0.19	0.02	−0.08
L⋅min^–1^	0.03	−0.11	−0.11
mL⋅kg^–0.67^⋅min^–1^	−0.12	−0.03	−0.10
%RUN-VO_2max_	−0.08	−0.72**	0.16
HF	0.10	−0.25	−0.15
TTE (s)	−0.25	0.30	−0.02
**ΔC_DP_ at LT**			
mL⋅kg^–1^⋅m^–1^	−0.02	0.19	−0.85**
mL⋅kg^–0.67^⋅m^–1^	0.01	0.16	−0.79**
**ΔMAS**			
m⋅min^–1^	−0.09	−0.21	−
km⋅h^–1^	−0.09	−0.21	−
**ΔLT**			
%DP-VO_2peak_	−0.09	0.24	−0.53**
HF	0.12	−0.09	−0.46*
VO_2_	−0.16	0.23	−0.49**
[La^–^_b_]	0.004	−0.10	−0.17
Speed (km⋅h^–1^)	−0.16	0.05	0.57**
**ΔStrength**			
Half squat	−0.20	−0.23	−0.01
Pull-down	−0.28	0.10	−0.20
**ΔPower**			
Half squat	0.30	−0.15	−0.17
Pull-down	0.05	0.13	−0.28
SJ	−0.24	0.42*	0.22
CMJ	−0.12	0.13	−0.12
CMJas	−0.11	−0.12	0.20

Values are correlation coefficients *r*. ΔBW, change in body-weight. Kg, kilograms. ΔTT_*DP*_, change in double poling time trial. ΔRUN-VO_2max_, change in maximal oxygen uptake in running. mL⋅kg^–1^⋅min^–1^, milliliters per kilogram bodyweight per minute. L⋅min^–1^, liters per minute. mL⋅kg^–0.67^⋅min^–1^, milliliters per kilogram raised to the power of −0.67 per minute. HR, heart rate. RER, respiratory exchange ratio. [La^–^_*b*_], blood lactate concentration. RPE, rate of perceived exertion. ΔDP-VO_2peak_, change in peak oxygen uptake in double poling. %RUN-VO_2max_, fractional utilization of RUN-VO_2max_ at DP-VO_2peak_. TTE, time to exhaustion. ΔC_*DP*_, change in oxygen cost of double poling at lactate threshold. ΔMAS, change in maximal aerobic speed. ΔLT, change in lactate threshold. VO_2_, oxygen uptake. Km, kilometers. H, hours. 1RM, one repetition maximum. SJ, squat jump. CMJ, counter-movement jump. CMJas, counter-movement jump with armswing. * *p* < 0.05 significant delta correlation. ** *p* < 0.01 significant delta correlation.

No delta correlations were observed between ΔTT_DP_ and any delta values of the physiological or training variables (Tables 5, 6). ΔMAS revealed strong significant correlations to ΔLT_v_ (*r* = 0.57, *p* < 0.01) and ΔC_DP_ (*r* = −0.85, *p* < 0.01). Δski specific training and ΔLIT_ski_ showed low significant correlations to ΔLT_v_ (*r* = 0.48, *p* < 0.01 and *r* = 0.45, *p* < 0.05, respectively), while ΔLIT_ski_ showed a low significant correlation to ΔC_DP_ (*r* = −0.41, *p* < 0.05).

### Sex Differences

**FIGURE 1:**
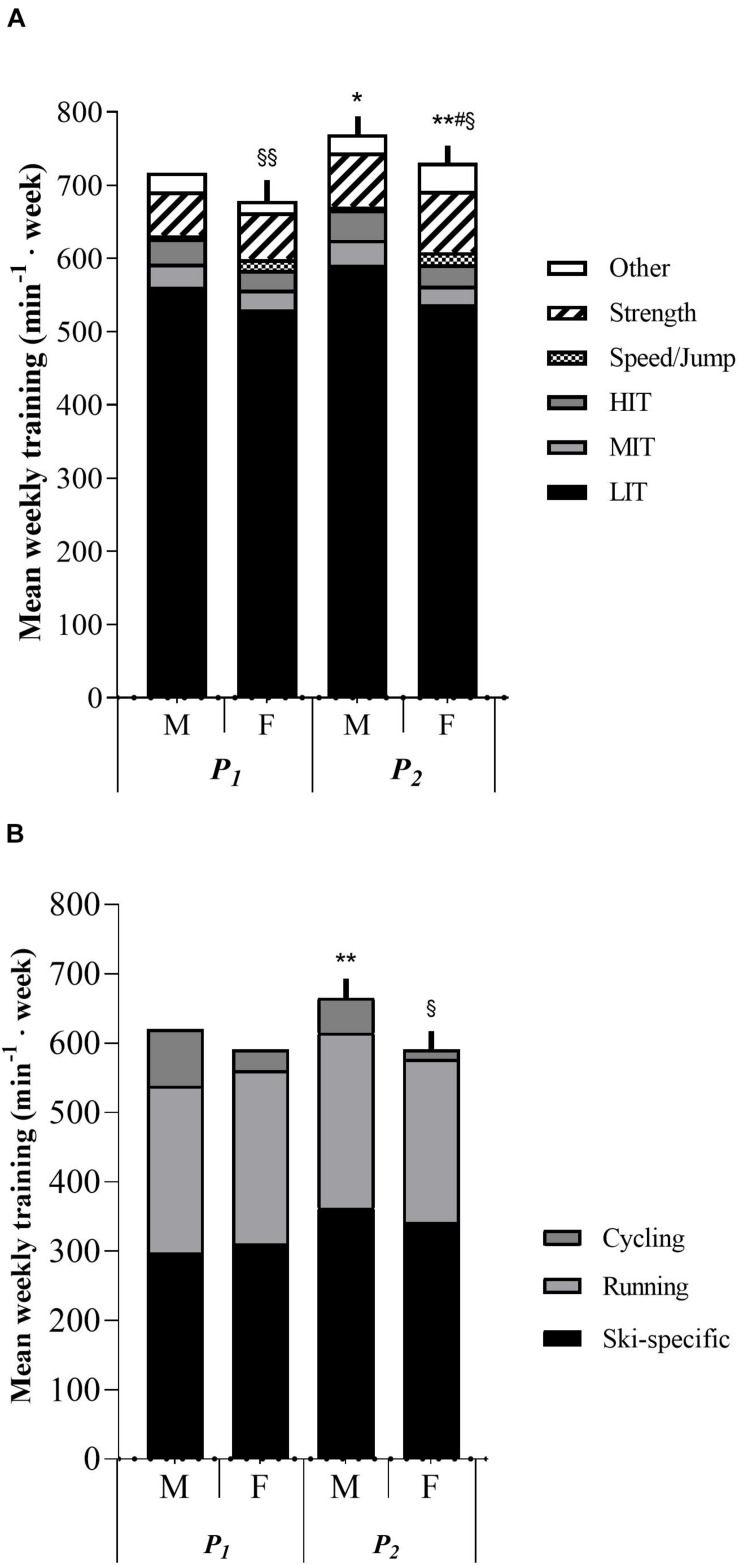
**(A)** Sex differences between males and females in training characteristics in minutes per week. M, males. F, females. *P*_1_, first training period from May to July. *P*_2_, second training period from August to October. LIT, low-intensity endurance training. MIT, moderate-intensity endurance training. HIT, high-intensity training. min^– 1^ week, minutes per week. *p < 0.05 significant difference from *P*_1_ in strength and other training. **p < 0.01 significant difference from *P*_1_ in strength training. ^#^*p* < 0.05 significantly different in delta values in other training from male value. ^§^ *p* < 0.05 significantly different from male value in speed/jump training volume. ^§§^ *p* < 0.01 significantly different from male value in speed/jump training volume. **(B)** Sex differences between males and females in endurance training mode in minutes per week. **p < 0.01 significantly different from *P*_1_ value in ski-specific training. ^§^ *p* < 0.05 significantly different from male value in cycling.

Male skiers trained significantly higher volumes than females 3 months before pre-tests (*p* < 0.05). Additionally, no statistical difference was observed in LIT, MIT, HIT, total endurance training, ski-specific training, running, strength training or other training between males and females in either *P*_1_ or *P*_2_. Males trained significantly more cycling in *P*_2_ (*p* < 0.05), and females trained significantly higher volumes of speed/jump training, both in *P*_1_ (*p* < 0.01) and *P*_2_ (*p* < 0.05). No statistical difference was observed in training progression throughout the 6-months period between males and females, except for other training (*p* < 0.05). Differences in training characteristics between males and females are shown in Figures 1A,B and Supplementary Table 4.

Significant sex differences in physiological and performance variables were observed at PRE-tests. Results from physiological and performance tests are presented in Supplementary Table 1. Males had on average 14.7% (*p* < 0.01, effect size = 2.28) better TT_DP_ performance, 19.3% (*p* < 0.01, effect size = 1.91) higher RUN-VO_2max_ (mL⋅kg^–1^⋅min^–1^), 19.3% (*p* < 0.01, effect size = 1.72) higher DP-VO_2peak_ (mL⋅kg^–1^⋅min^–1^), 9.1% (*p* < 0.01, effect size = 0.97) better C_DP_ (mL⋅kg^–1^⋅m), 30.2% (*p* < 0.01, effect size = 2.04) higher LT_v_ and a 32.3% (*p* < 0.01, effect size = 2.16) higher MAS than females at baseline. In addition, males were 21.3% (*p* < 0.01, effect size = 1.22) and 30.5% (*p* < 0.01, effect size = 1.86) stronger than females in half squat and pull-down, respectively, and displayed 34.7% (*p* < 0.01, effect size = 1.37) and 45.9% (*p* < 0.01, effect size = 1.37) higher power values in half squat and pull-down, respectively. No significant gender differences were apparent in HR, RER, [La^–^]_b_ or RPE in running or double-poling, %RUN-VO_2max_ or LT%, at baseline (all effect sizes <0.7).

**FIGURE 2:**
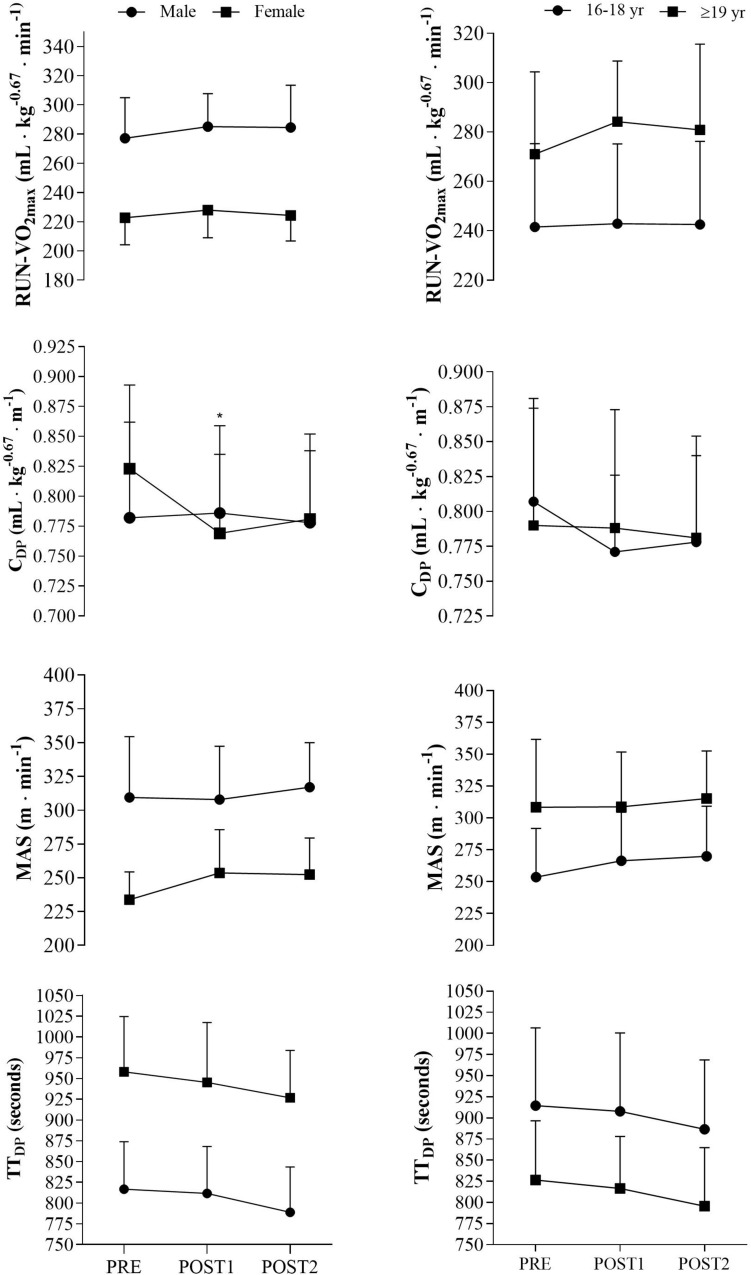
Sex and age differences in key physiological and performance variables. RUN-VO_2max_, maximal oxygen uptake in running. mL⋅kg^– 0.67^⋅min^– 1^, milliliters per kilogram raised to the power of –0.67 per minute. C_DP_, oxygen cost of double poling at lactate threshold. mL⋅kg^– 0.67^⋅m^– 1^, milliliters per kilogram raised to the power of –0.67 per meter. MAS, maximal aerobic speed. m⋅min^– 1^, meter per minute. TT_DP_, time trial performance in double poling. *p < 0.05 significant different from male delta value.

No sex differences were observed in physiological or performance adaptations from PRE to POST2, except for [La^–^]_b_ in RUN-VO_2max_ (*p* < 0.05, effect size = 0.93). From PRE to POST1, only RER_*RUN*_ (*p* < 0.05, effect size = 0.88), C_DP_ (*p* < 0.05, effect size = 0.77), and LT% (*p* < 0.05, effect size = 0.91) changed significantly different between males and females. However, no gender differences were observed in physiological or performance adaptations from POST1 to POST2. Training adaptations for males and females in key physiological variables are presented in Figure 2.

### Age-Group Differences

**FIGURE 3:**
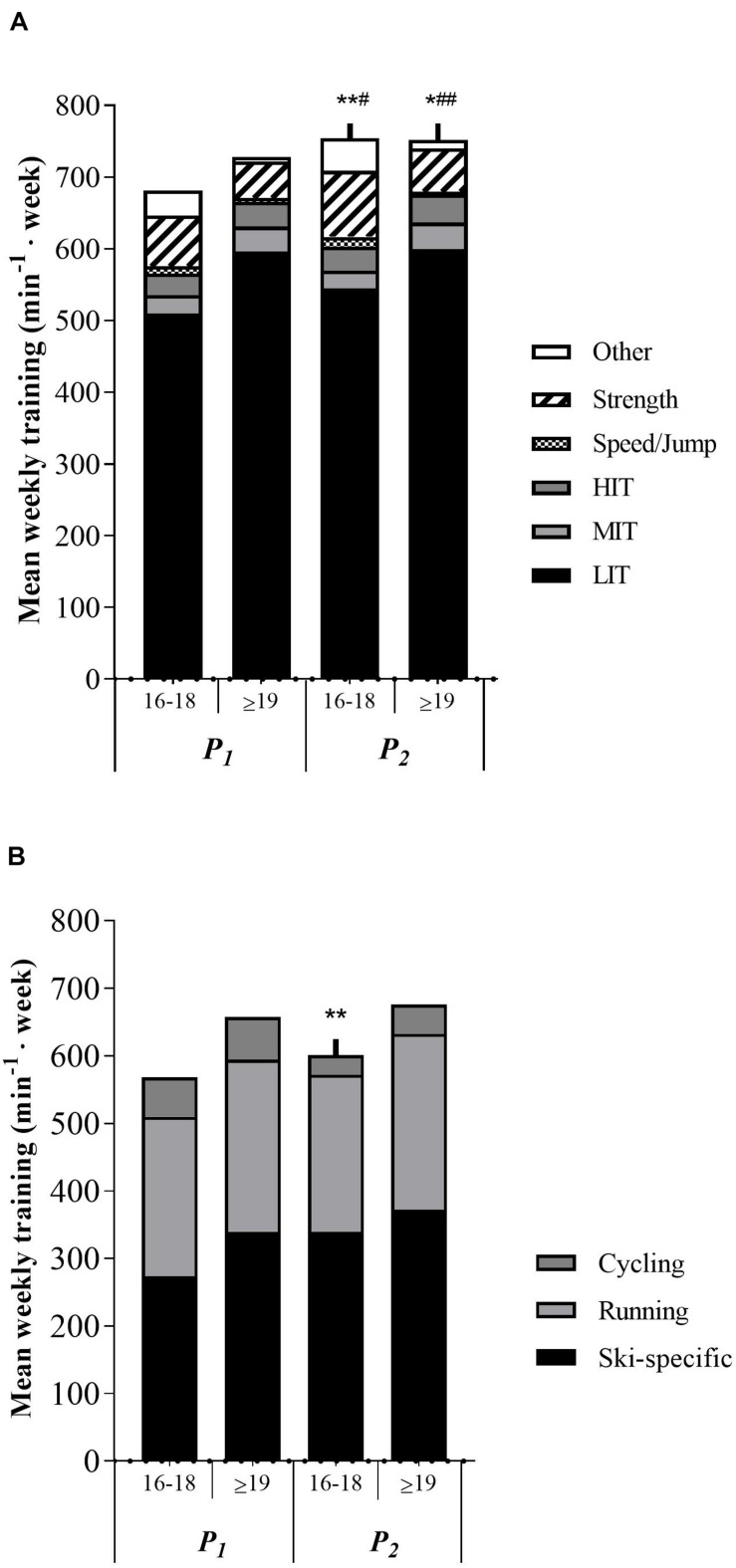
**(A)** Age differences in training characteristics in minutes per week. *P*_1_, first training period from May to July. *P*_2_, second training period from August to October. LIT, low-intensity endurance training. MIT, moderate-intensity endurance training. HIT, high-intensity training. min^– 1^ week, minutes per week. *p < 0.05 significantly different from *P*_1_ in strength training. **p < 0.01 significantly different from *P*_1_ in total training volume and strength training. ^#^*p* < 0.05 significantly different from ≥19 years value in speed/jump training. ^##^*p* < 0.01 significantly different from 16 to 18 years value in strength and other training. **(B)** Age differences in endurance training mode in minutes per week. **p < 0.01 significantly different from *P*_1_ in ski-specific training.

No age differences were observed in total training volume 3-months before PRE. Total MIT volume was significantly higher in the ≥19 years group (*p* < 0.05) in both *P*_1_ and *P*_2_, while other training volume was significantly lower in the same group compared to the 16–18 years group. Speed/jump and strength training was significantly lower in the ≥19 years group in *P*_2_ (*p* < 0.05 and *p* < 0.01, respectively), while no difference was apparent in *P*_1_. No training differences between age groups were displayed in total training volume, LIT, HIT, ski-specific training, running, or cycling during the whole training period. No age group differences were observed in delta training values throughout the whole period. Training characteristics for the two age groups are presented in Figures 3A,B and Supplementary Table 5.

Results from physiological and performance tests among age-groups are presented in Supplementary Table 2. At PRE the ≥19 years group had a 9.6% (*p* < 0.01) better TT_DP_ performance, 12.0% (*p* < 0.05) higher DP-VO_2peak_ (mL⋅kg^–1^⋅min^–1^), 7.3% (*p* < 0.05) better C_DP_, 23.4% (*p* < 0.01) higher LT_v_ and 21.6% (*p* < 0.01) higher MAS than the younger skiers. In addition, the oldest skiers were significantly stronger in half squat (15.9%, *p* < 0.05) and pull-down (26.5%, *p* < 0.01), and had higher maximal power values both in half squat (35.1%, *p* < 0.01) and pull-down (41.5%, *p* < 0.01). No differences were observed between age groups in RUN-VO_2max_ (mL⋅ kg^–1^ ⋅ min^–1^), RER, [La^–^]_b_, %RUN-VO_2max_ or LT%.

No differences in delta values was observed between age groups from PRE to POST2, except for BW (*p* < 0.05, effect size = 0.94), [La^–^]_b_ in running (*p* < 0.05, effect size = 0.92), LT_v_ (*p* < 0.05, effect size = 0.87) and power in half squat (*p* < 0.05, effect size = 0.88). Additionally, differences in delta values were observed in RUN-VO_2max_ (mL⋅kg^–1^⋅min^–1^, *p* < 0.05, effect size = 0.87) and power in half squat (*p* < 0.01, effect size = 1.22) from PRE to POST1. No differences were observed in physiological and performance adaptations from POST1 to POST2. Training adaptations in key variables for the two age-groups are presented in Figure 2.

### Impact of the Selected Genes

All polymorphisms were successfully genotyped, and were at Hardy-Weinberg equilibrium (*p* > 0.05). Genotype frequencies are displayed in Table 7. Minor allele frequencies (MAF) for the genotyped polymorphisms were as follows: 53% for *ACTN3*, 41% for *ACE* and *IL6*, 40% for *ACSL1*, 38% for *PPARGC1A*, 19% for *PPARA* and 9% for *PPARG* polymorphism.

**TABLE 7:** Genotype distributions for selected genes (*n* = 29).

***ACTN3***		***ACE***		***ACSL1***		***IL6***	
RR	7 (24.1)	DD	9 (31.0)	GG	11 (37.9)	CC	7 (24.1)
RX	13 (44.8)	ID	16 (55.2)	GA	13 (44.8)	AC	20 (69.0)
XX	9 (31.0)	II	4 (13.8)	AA	5 (17.2)	AA	2 (6.9)

***PPARGC1A***		***PPARG***		***PPARA***			

CC	8 (27.6)	GG	24 (82.8)	GG	20 (69.0)		
CT	20 (69.0)	CG	5 (17.2)	CG	7 (24.1)		
TT	1 (3.4)	CC	0 (0.0)	CC	2 (6.9)		

*Values are presented as *n* with percentage genotype distribution in parenthesis. Genotypes are *ACTN3* R577X, *ACE* I/D, *ACSL1* rs6552828, *IL6* rs1474347, *PPARGC1A* rs819267, *PPARG* rs1801282, and *PPARA* rs4253778 polymorphisms.*

Key physiological and performance results among genotypes in *ACTN3* and *ACE* at baseline is presented in Table 8. All genotype and allele data is presented in Supplementary Table 6. There were no differences in physiological and performance results between the three *ACTN3* genotypes when analyzing the 29 included skiers. The same picture was shown when analyzing all successfully genotyped participants (*n* = 40), except for a significantly higher DP-VO_2peak_ in the RX genotype compared to the RR genotype (Supplementary Table 8). When testing X allele carriers compared to the RR genotype, DP-VO_2peak_ (mL⋅kg^–1^⋅min^–1^), both independent of- and corrected for gender, was, respectively, 12.4 and 8.8% higher (*p* < 0.05, effect sizes >0.80).

**TABLE 8:** Physiological baseline results divided by *ACTN3* and *ACE* genotypes.

	***ACTN3***		
**Genotype (N)**	**RR (7)**	**RX (13)**	**XX (9)**
**Independent of gender**			
RUN-VO_2max_	237.9 ± 43.9	259.2 ± 36.2	261.1 ± 28.7
DP-VO_2peak_	201.5 ± 32.2	226.1 ± 36.9	225.6 ± 24.6
C_DP_	0.796 ± 0.047	0.779 ± 0.082	0.829 ± 0.066
1RM half squat	113.6 ± 19.5	120.2 ± 21.6	127.2 ± 24.5
1RM pull-down	89.3 ± 19.7	84.2 ± 16.7	90.6 ± 14.7
**Corrected for gender**			
RUN-VO_2max_	267.1 ± 22.9	285.7 ± 25.2	273.6 ± 22.4
DP-VO_2peak_	227.4 ± 21.5	249.3 ± 28.7	236.0 ± 10.9
C_DP_	0.773 ± 0.042	0.760 ± 0.074	0.820 ± 0.064
1RM half squat	126.1 ± 14.7	131.7 ± 20.2	132.8 ± 24.7
1RM pull-down	102.6 ± 13.2	94.8 ± 16.5	95.1 ± 7.8

	***ACE***		
**Genotype (N)**	**DD (9)**	**ID (16)**	**II (4)**

**Independent of gender**			
RUN-VO_2max_	254.848.4	256.329.5	247.638.6
DP-VO_2peak_	220.9 ± 44.0	222.6 ± 28.9	207.3 ± 24.3
C_DP_	0.755 ± 0.070	0.812 ± 0.067	0.844 ± 0.051
1RM half squat	121.4 ± 27.8	122.7 ± 16.1	112.5 ± 30.7
1RM pull-down	92.2 ± 16.4*	89.4 ± 15.2	68.8 ± 11.1
**Corrected for gender**			
RUN-VO_2max_	278.1 ± 29.0	273.9 ± 23.7	290.1 ± 13.1
DP-VO_2peak_	240.8 ± 24.1	238.4 ± 27.0	244.2 ± 11.0
C_DP_	0.738 ± 0.066	0.799 ± 0.065	0.811 ± 0.035
1RM half squat	131.3 ± 23.2	130.8 ± 17.9	128.5 ± 25.9
1RM pull-down	102.7 ± 9.8*	96.81 ± 4.8	83.2 ± 3.3

Values are mean ± SD. RUN-VO_2max_, maximal oxygen uptake in running expressed in milliliters per kilogram bodyweight raised to the power of −0.67 per minute. DP-VO_2peak_, peak oxygen uptake in double poling expressed in milliliters per kilogram bodyweight raised to the power of −0.67 per minute. C_*DP*_, oxygen cost of double poling expressed in milliliters per kilogram bodyweight raised to the power of −0.67 per meter. 1RM, one repetition maximum expressed in kilograms. * *p* < 0.05 significantly different from *ACE* II genotype.

For the *ACE* I/D genotypes, individuals with the DD genotype displayed an 18.4% better C_DP_ (mL⋅kg^–0.67^⋅m^–1^) compared to those with the II genotype (*p* < 0.01, effect size = 1.42). However, corrected for gender this difference was no longer significant. Individuals with DD genotype also displayed significantly higher values in 1RM pull-down from the II counterparts, both dependent (34%, *p* = 0.05, effect size = 1.67) and independent (23.4%, *p* < 0.05, effect size = 2.66) of gender (Table 8). Although the same picture was apparent, all significant associations disappeared when analyzing all 40 successfully genotyped participants (Supplementary Table 8). When comparing II genotypes to D carriers we detected a 9.2% higher RUN-VO_2max_ (mL⋅kg^–1^⋅min^–1^), when corrected for gender (*p* < 0.05, effect size = 1.14). Overall, D allele carriers were 27.2% stronger than individuals with the II genotype (*p* < 0.05), and the association remained significant when corrected for gender (17.4%, *p* < 0.01, effect size = 1.27).

There were no significant associations between genotypes of the *ACSL1* rs6552828 polymorphism and physiological variables. However, when corrected for gender, an 8.4% difference was observed in RUN-VO_2max_ (mL⋅kg^–1^⋅min^–1^ and mL⋅kg^–0.67^⋅min^–0.67^; *p* < 0.05) between A allele carriers compared to the GG genotype. As to the *PPARGC1A* rs8192678, the highest RUN-VO_2max_ (mL⋅kg^–1^⋅min^–1^, L⋅min^–1^ and mL⋅kg^–0.67^⋅min^–1^) was exhibited by the only individual with TT. Significant differences were observed in RUN-VO_2max_ (*p* < 0.05), DP-VO_2peak_ (*p* < 0.05) and 1RM pull-down (*p* < 0.05) between genotypes and allele carriers in *PPARA* rs4253778, *IL6* rs1474347 and *PPARG* rs1801282 independent of gender. However, when corrected for gender, these differences disappeared.

## Discussion

### Main Findings

This is the first study to investigate effects of age, sex, selected genes and training on physiological and performance characteristics and adaptations in well-trained cross-country skiers. Throughout the 6 months period, the skiers displayed no differences in relative distribution of training intensity, although the total training volume and relative amount of ski-specific training increased. This led to no significant differences in physiological and performance variables. Neither was there observed any differences between groups in training adaptations throughout the 6 months training period. At baseline, the male skiers trained more than the female skiers did, but with approximately the same distribution regarding training modalities and intensity zones. We did not detect any major differences in physiological or performance variables based on genotypes.

### Training Characteristics

The participants in the present study were all well-trained and competitive skiers, although not international elite athletes. All skiers competed at national events, and some skiers competed at Scandinavian or international long-distance events (i.e., Vasaloppet and Marcialonga). Thus, all athletes planned and performed their training with a goal of developing better performance capacity. Compared to training data from elite cross-country skiers (Losnegaard et al., 2013; Tønnessen et al., 2014; Sandbakk et al., 2016; Solli et al., 2017, 2018), most of the participants displayed lower training volumes. This may partly be explained by the fact that the skiers in the present study had to perform their daily training beside other work duties, studies or family obligations. The national level skiers in the study of Sandbakk et al. (2016) are thus more comparable to these participants. Secondly, approximately 50% of the skiers in the present study were still 16 to 18 years, and their total training volume was not statistically different from the older (≥19 years) skiers. This may be because the younger skiers were attending different skiing high schools, were training was an incorporated part of the school schedule. In previous studies, total training volume of younger skiers were lower than in older skiers (Sandbakk et al., 2010, 2013, 2016; Losnegaard et al., 2013; Solli et al., 2017).

The relative distribution of training intensity in the present study was in line with general recommendations for cross-country ski training (Sandbakk and Holmberg, 2017). Cross-country skiers are generally recommended to train with high amounts of LIT and low to moderate amounts of MIT and HIT during the preparation period (May to October). In the study of Sandbakk et al. (2016) national-class cross-country skiers performed on average 90% of their total endurance training at <81% of HR_max_, 4% at 82–87% HR_max_ and 6% at 88% of HR_max_ or higher from May to October. The skiers in the present study displayed, respectively 90, 5, and 5% at the same intensity zones from May to July, showing almost exactly the same intensity distribution from August to October (88, 5, and 6%, respectively). However, different training quantification methods were used in the present study compared to Losnegaard et al. (2013) and Sandbakk et al. (2016). In the present study, training was registered as “time in zone,” meaning the exact time in each intensity zone regardless of how the training may have been planned. In Losnegaard et al. (2013) and Sandbakk et al. (2016), the session-goal approach was used, meaning that average HR during either continuous workouts or interval bouts is used to determine the intensity distribution during sessions. This makes the training data difficult to compare with the results in the present study, at least in the higher intensity zones. Sylta et al. (2014) has suggested a conversion factor of 1:3 for comparison of “time-in-zone” training data to session-goal training data for HIT training. By use of this factor on training data from Losnegaard et al. (2013) and Sandbakk et al. (2016), the HIT training should be only a third of what was reported. The study of Tønnessen et al. (2014) shows more comparable “time in zone” data for MIT and HIT in Olympic-medal winning skiers as the skiers in the present study. However, their study showed higher volumes of both HIT and total training volume compared to the present study.

In the present study, the skiers displayed almost no progression in training volume or relative distribution of training intensity during the 6 months period. These findings are in contrast to the study of Sandbakk et al. (2016), where the national level skiers showed a linear increase in training volume from May to September, generally due to higher volumes of LIT. However, the elite international skiers in the studies of Sandbakk et al. (2016) and Losnegaard et al. (2013) showed progression more similar to the skiers in the present study from May to October, but with an increase in HIT and a decrease in LIT during the competitive season, i.e., November to April. The latter may also be the case for the skiers in the present study, but training during the competitive season was not investigated here.

The skiers in the present study performed on average 50% of their total endurance training on ski-specific training, mainly roller skiing. The other half was devoted for the most part to running. These volumes and relative distribution of ski-specific vs. unspecific training are in close agreement with previous studies on cross-country skiers, both non-elite and elite (Losnegaard et al., 2013; Tønnessen et al., 2014; Sandbakk et al., 2016; Solli et al., 2017). Ski-specific training may target the ski-specific aerobic capacity, work economy and technical factors better than general endurance training (Johansen et al., 2020). The skiers added approximately 60 min per week of ski-specific training from *P*_1_ to *P*_2_, while running was held relatively constant throughout the whole training period. This may be a result of a desire to elevate ski-specific capacities (i.e., work economy or technical factors) closer to the competitive season, in line with training characteristics from Losnegaard et al. (2013) and Sandbakk et al. (2016).

The relative distribution of strength and speed/jump training was comparable to the training volumes observed previously (Losnegaard et al., 2013; Tønnessen et al., 2014). This is in line with the increased focus on the upper- and lower body strength and speed in modern cross-country skiing (Sandbakk and Holmberg, 2017; Sunde et al., 2019).

### Training Adaptations

The 6 months of training from May to October led to no significant improvements in physiological and performance variables for the skiers in the present study. Since the training volume and intensity distribution was almost constant throughout the study period, this was no surprise. It is still noteworthy that junior- and sub-elite cross-country skiers that train a total of approximately 300 h from May to October show no improvements in physiological factors and only minor improvements in performance. On the other hand, the training performed was sufficient to maintain physiological and performance variables throughout the study period.

Strong correlations (*p* < 0.01) were found at baseline between TT performance and MAS (*r* = −0.79), DP-VO_2peak_ (*r* = −0.83) and 1RM pulldown (*r* = −0.72). When corrected for gender all these correlations were still significant. This is in line with other studies examining performance determining factors in endurance sports, i.e., running, cycling and cross-country skiing (Pate and Kriska, 1984; Ingjer, 1991; di Prampero, 2003; Støren et al., 2013; Sunde et al., 2019; Johansen et al., 2020). Several studies have also observed better performance after improved MAS (Støren et al., 2008, 2012; Sunde et al., 2010; Johansen et al., 2020) or improved maximal strength (Hoff et al., 2002; Støren et al., 2008; Sunde et al., 2010). However, no significant relationships between changes in TT_DP_ performance and changes in physiological variables were found in the present study.

The 3.3% non-significant improvement in TT performance in the present study is approximately half of that reported in Losnegaard et al. (2013). That study observed a 6% improvement from June to October in a 1000-meter TT in V2 skating in elite cross-country skiers, despite no improvements in VO_peak_ in V2 skating. However, C in V2 skating was significantly improved suggesting that the improvement in performance was due to an improvement in MAS in that study. Losnegaard et al. (2013) also explained the better performance by increased anaerobic capacity, measured as ΣO_2_-deficit. Anaerobic capacity was not measured directly in the present study. However, compared to the 1000 m TT used in Losnegaard et al. (2013) the anaerobic capacity should be of lesser importance in the 5.6 km TT used in the present study.

Overall aerobic capacity (RUN-VO_2max_) and specific (DP-VO_2peak_) aerobic capacity was not improved significantly from May to October in the present study. These findings are in line with studies investigating training patterns and development in VO_2max_ in well-trained or elite cross-country skiers maintaining similar training routines (volume and intensity distribution) over longer periods (Rusko, 1987; Ingjer, 1992; Jones, 1998; Gaskill et al., 1999; Losnegaard et al., 2013; Solli et al., 2017). Further improvements of extremely high VO_2max_ in elite endurance athletes have shown to be challenging. Compared to their elite counterparts, the skiers in the present study had approximately 20% lower aerobic capacities (Tønnessen et al., 2015; Sandbakk and Holmberg, 2017). This suggests that the potential for further improvements should be higher for the skiers in the present study, at least for the younger skiers. There is much evidence supporting that HIT may effectively improve VO_2max_, both in recreational and elite endurance athletes (Nilsson et al., 2004; Støren et al., 2012; Sandbakk et al., 2013; Rønnestad et al., 2014, 2016; Johansen et al., 2020). However, these interventional studies include longer or shorter periods of higher amounts of HIT, and lower total training volume. Stöggl and Sperlich (2014) reported superior adaptations in well-trained endurance athletes after 9 weeks of polarized training (56% LIT, 3% MIT, and 26% HIT) and HIT (43% LIT, 0% MIT, and 57% HIT) in VO_2max_, compared to training models with no training at HIT intensities and higher training volumes. This is well in line with the studies of Støren et al. (2012) and Gaskill et al. (1999), where endurance athletes experienced great improvements with a training program with higher amounts of HIT, with the same, or reduced total training volumes. Thus, we may speculate that more HIT training during pre-season may be crucial for further development of aerobic capacity in junior- and sub-elite cross-country skiers.

No statistically significant improvements in C_DP_ were observed. However, like most of the other physiological variables, a slightly better average C_DP_ was seen, although not significant. Losnegaard et al. (2013) reported improved C from June to October in elite cross-country skiers and this could be due to the increased ski-specific training. A significant correlation was also observed between change in total ski-specific training and ΔC_DP_ in the present study, suggesting that adaptation is specifically to the movement patterns used in training (Scrimgeour et al., 1986; McMillan et al., 2005; Johansen et al., 2020). Previous studies have reported improved C after MST in both running (Støren et al., 2008), cycling (Sunde et al., 2010) and cross-country skiing (Hoff et al., 2002; Østerås et al., 2002). However, this relationship was not observed for the whole group in the present study, since MST and thus C_DP_ did not change during the 6 month period.

Several previous studies have reported no training adaptations in LT in % of VO_2max_ after shorter or longer periods of endurance or strength training (Helgerud et al., 2001, 2007; Støren et al., 2008, 2012; Sunde et al., 2010; Rønnestad et al., 2014). This is in line with results in the present study, since the skiers had almost exactly the same LT% at all test points. The present study also showed a strong correlation at baseline between MAS and LT_v_ (*r* = 0.93, *p* < 0.01) indicating a close relationship, which have previously been reported (Støren et al., 2014; Sunde et al., 2019). Consequently, to elevate LT_v_ skiers should aim to improve MAS (VO_2max_ and C).

### Sex Differences

Males had higher training volumes than females preceding the baseline tests in the present study. However, from May to October no significant sex differences were observed in total training volume, relative intensity distribution, endurance training, ski-specific training, strength training or other training. These training characteristics are in line with the findings in elite cross-country skiers from Solli et al. (2018), where males tended to train more in total than females throughout a whole year (∼ 90 h), although not significant. In Solli et al. (2018), strength and speed training was similar for males and females, as observed in the present study regarding strength training. However, in the present study the amount of speed and jump training was four times higher in females than males.

Males displayed significantly higher values than females in RUN-VO_2max_ (19%), DP-VO_2peak_ (19%), and MAS (32%), had better C_DP_ (9%) and TT_DP_ (15%) at baseline in the present study. These sex differences are in line with previous results (Sandbakk et al., 2014; Andersson et al., 2019; Sunde et al., 2019). Since MAS is the product of DP-VO_2peak_ and C_DP_ it was no surprise that the sum of sex differences in these two variables equalled almost exactly the difference seen in MAS. The gender difference in TT_DP_ seemed to correspond to the 32% difference in MAS. This is further supported by the correlation between MAS and TT_DP_ (*r* = −0.58, *p* < 0.01) at baseline corrected for gender.

Interestingly, the sex differences in DP-VO_2peak_ was the same as in RUN-VO_2max_ in the present study. Sandbakk et al. (2014) and Hegge et al. (2016), found the sex differences to be larger with increased contribution of upper-body musculature, i.e., larger in DP-VO_2peak_ than in RUN-VO_2max_. Regarding 1RM strength variables, the gender differences were larger in 1RM pull-down (30%) compared to 1RM half-squat (21%) in the present study. These sex differences in 1RM strength are in line with previous results (Sandbakk et al., 2014; Sunde et al., 2019).

The sex differences at baseline in the present study were maintained in TT_DP_, RUN-VO_2max_, and DP-VO_2peak_ from May to October due to no significant differences in training progression between males and females in this period. This may suggest that males and females do not differ in physiological and performance adaptations to a similar training pattern, which is in line with previous studies (Astorino et al., 2011; Støren et al., 2017; Varley-Campbell et al., 2018). However, sex difference in C_DP_ declined significantly from PRE to POST1, due to a significantly improved C_DP_ in females while the males maintained their pre-values. This may be explained by the lower training volumes in females 3-months prior to pre-test, resulting in a greater sex difference in C_DP_ at PRE. From May to July, males and females trained more similar, at least in terms of ski-specific training, and this may have reduced the initial gap. Another explanation for the improved C_DP_ observed in females, may be the relationship observed earlier between improved maximal strength and improved C in running, cycling and cross-country skiing (Hoff et al., 2002; Støren et al., 2008; Sunde et al., 2010). In the present study, a significant correlation was observed between Δ1RM pull-down and ΔC_DP_ (*r* = −0.60, *p* < 0.05) in the female skiers, which supports this explanation. However, further improvements in C_DP_ was not observed in females or males from August to October.

### Age-Related Differences

To the best of our knowledge, no studies have investigated age-related differences in training characteristics and training adaptations between younger (junior athletes) and older skiers (senior athletes). In the present study, the age groups did not differ significantly in total training volume 3-months prior to pre-test. No differences were apparent in total training volume or in LIT, HIT, ski-specific training, running, cycling, strength or speed/jump training during the preparation period from May to October. The only difference between 16 and 18 years old compared to ≥19 years old was the amount of MIT, where the average difference were ∼ 20 min per week. Compared to other studies examining either junior- or adult skiers, the 16–18 years old skiers in the present study show similar training volumes as seen in previous studies on junior athletes, however, with a slightly lower amount of MIT and HIT (Sandbakk et al., 2011, 2013). However, the older skiers had lower training volumes compared to age-matched adult elite cross-country skiers (Losnegaard et al., 2013; Tønnessen et al., 2014; Sandbakk et al., 2016; Solli et al., 2017).

From May to October, the young and adult skiers did not differ significantly in training progression. The oldest skiers displayed almost no progression in all training variables, throughout the study period. This is in accordance to earlier observations of training progression in adult elite cross-country skiers (Losnegaard et al., 2013; Sandbakk et al., 2016).

At baseline, the adult skiers were 15% heavier than the younger skiers. A significant age-related difference was also apparent in TT_DP_ (10.6%), which was followed by a 17.8% difference in MAS. Corrected for age, MAS showed a strong correlation to TT_DP_ at baseline (*r* = −0.77, *p* < 0.01). The difference in MAS was almost exactly the same as in LT_v_, supporting that DP-VO_2peak_ and C_DP_ are the main predictors for LT_v_. The age difference in MAS is a consequence of the 10.7% difference in DP-VO_2peak_ and the 7.9% difference in C_DP_. The age difference in RUN-VO_2max_ and DP-VO_2peak_ may be attributed to incomplete development of the cardiac system and muscle mass in the younger skiers still in puberty (Rusko, 1992). Additionally, the lower number of years of training in the young skiers may be an explanation for the observed difference. The difference in C_DP_ may also be a result of less training years and experience in the younger skiers. In addition, the adult skiers had 21% higher 1RM pull-down than the younger skiers. Stronger skiers have shown to have higher peak forces in DP, lower DP frequency and shorter contact time (Sunde et al., 2019). However, all these age differences should be handled with great caution as they are most probably due to the sex differences in the two age groups. When analyzing age differences in males and females separately in the two groups, young and adult females differed in the same physiological and performance variables observed for the whole group, except for C_DP_ and strength variables. For the males, almost every significant age-related difference disappeared, except for 1RM pull-down and DP-VO_2peak_ in absolute values.

### Effect of Selected Genes

We did not detect any major effects for the selected genes on physical and performance variables at baseline. Based on the low number of participants and the expected influence by single genes, this was not unexpected. However, we did find some minor effects.

In the present study the common *ACTN3* R577X, X allele carriers demonstrated higher DP-VO_2peak_ than participants with the RR genotype at PRE. This is in accordance to Pimenta et al. (2013) that observed that soccer players with the XX genotype had the highest VO_2max_. According to Yang et al. (2003) the X allele is overrepresented among endurance athletes, especially females. The importance of the advantageous allele is also likely dependent on the performance level (Eynon et al., 2012). However, others have not been able to confirm this (Papadimitriou et al., 2018). The X allele frequency in the present study was slightly higher (44 vs. 53%) among athletes than the general population from the same geographical area (Goleva-Fjellet et al., 2020). For the *ACE* gene, skiers with the II genotype exhibited higher RUN-VO_2max_ compared with carriers of the D allele. However, participants with DD genotype demonstrated ∼15% better C_DP_ and had ∼28% higher 1RM pull-down compared to the II genotype. The observed superior C_DP_ among skiers with the DD genotype could be explained by gender differences in 1RM (Sunde et al., 2019). The I allele frequency among the cross-country skiers included in the present study was 14.6% higher than in a Norwegian cohort from the same geographic region (Goleva-Fjellet et al., 2020).

For the *ACSL1* rs6552828, the A allele carriers had 8.4% higher RUN-VO_2max_ (mL⋅kg^–1^⋅min^–1^ and mL⋅kg^–0.67^⋅min^–1^; *p* < 0.05) compared to the GG genotype. A relatively large effect of 6% on the training response of VO_2max_ have been reported previously with the carriers and the common G allele exhibiting larger increase than the homozygotes of the less common A allele (Bouchard et al., 2011). The differences between the findings in the present study and the study of Bouchard et al. (2011) might be due to the different study population profiles, i.e., highly trained athletes vs. sedentary adults, respectively. The latter group have a larger potential of increasing their VO_2max_ as a result of standardized exercise-training programs compared to athletes. The present study did not measure a significant increase in the VO_2max_ throughout the testing period.

All associations between *PPARGC1A* rs8192678 and physiological and performance variables dissapeared when correcting for gender. The C allele (Gly) have been suggested to be an elite status endurance allele favorable to athletic ability (Tharabenjasin et al., 2019; Petr et al., 2020). Homozygotes of the C allele are generally more responding aerobic training compared to the T allele (Ser) (Petr et al., 2018). In the present study, only a single male participant possesed the least favorable genotype for endurance performance, i.e., TT. Despite possessing an unfavorable genotype to endurance performance, he demonstrated the highest RUN-VO_2max_ of all participants. This points at carefullness when interpreting physiological performance based on single genes.

No significant associations were found for either of the following polymorphisms in the present study when corrected for gender: *PPARA* rs4253778, *IL6* rs1474347, and *PPARG* rs1801282. For muscle function and jumping capacity, this is well in line with previous findings in other sports, at least for the *PPARA* rs4253778 polymorphism (Stastny et al., 2019). However, Stastny et al. (2019) found significant associations to other muscle parameters, such as reactive muscle index.

Despite previous findings on the effects of the *ACE* I/D and the *ACTN3* R577X polymorphisms on athletic ability and trainability, the impact of these are not strong enough predictors to determine the athletic ability (Venezia and Roth, 2019). Results from the present study confirms that genotype frequencies for the two most investigated and replicated polymorphisms (i.e., *ACE* I/D and *ACTN3* R577X) among the cross-country skiers were similar to those from a large general Scandinavian cohort (Goleva-Fjellet et al., 2020). Furthermore, there was the case of the one skier that possessed the least favorable endurance genotype for the *PPARGC1A* SNP, but still demonstrated the highest VO_2max_. These findings may indicate that possessing the optimal alleles of the different polymorphisms may be beneficial for endurance performance, but it is not critical for the athletic ability (Venezia and Roth, 2019; Petr et al., 2020). This may be especially true for athletes competing at a national level compared to world-class elite athletes (Eynon et al., 2012; Papadimitriou et al., 2016). However, the results from the present study should be treated with some caution due to the limited sample size. Some genotypes within the selected genes were either not present or only apparent in 1–2 participants, and may therefore influence our results. The material is thus prone to false negative results (type 2 errors), and we can only state that there were minor associations between some genotypes and physiological variables in our cohort of 29 skiers. This should be taken into account when interpreting the genetic results from the present study. Also, these athletes were already well trained and could be argued to not represent a good sample population to detect associations between genotype variants and physiological or performance characteristics.

### Practical Implications

In the present study, maintaining the same training intensity distribution, and only increase total training volume was not sufficient to further improve aerobic capacity and cross-country skiing performance significantly throughout 6 months of training. This suggests that training programs with the same training intensity distribution, only differing in training volume, may not ensure optimal development of each individual skier independent of age and sex (Gaskill et al., 1999). For the individual well trained athlete, substantial changes in training volume and training intensity distribution could be necessary to facilitate further improvements, as observed in earlier studies (Gaskill et al., 1999; Støren et al., 2012; Bratland-Sanda et al., 2020). This is important knowledge for trainers of talented cross-country skiers that have faced stagnation.

An interesting finding in the present study is that our cohort of skiers did not differentiate genetically in two of the most investigated polymorphisms in association to athletic ability compared to a general Scandinavian cohort from the same geographical area. This may suggest that one might be able to reach a high national level in cross-country skiing without having the optimal genotypes in selected genes, with sufficient and individualized training.

## Conclusion

Sex and age did influence physiological and performance variables at baseline, but did not influence training adaptations. Since the skiers in the present study did not display major changes in training, it was no surprise that no adaptations occurred in physiological or performance variables either. The genotype variants of selected genes were not critical determinants for physiological and performance variables in national and sub-elite cross-country skiers in the present study.

## Data Availability Statement

Restrictions apply to the datasets: the datasets presented in this article are not readily available due to the Norwegian legislation regarding the publication of genetic data. Requests to access the datasets should be directed to the corresponding author.

## Ethics Statement

The studies involving human participants were reviewed and approved by Regional Ethics Commitee of South-Eastern Norway, Telemark, Norway. Written informed consent to participate in this study was provided by the participants’ legal guardian/next of kin.

## Author Contributions

J-MJ, SG-F, ØS, AS, MS, and JH all participated significantly in the planning and design of the study, as well as the data analyzing and the writing of the article. J-MJ, AS, ØS, SG-F, LG, LS, BF, and MS participated in the data collection. LG, LS, and BF also participated in the writing of the article. All authors read and approved the manuscript.

## Conflict of Interest

The authors declare that the research was conducted in the absence of any commercial or financial relationships that could be construed as a potential conflict of interest.
