# Supplementary material for: No Change – No Gain; The Effect of Age, Sex, Selected Genes and Training on Physiological and Performance Adaptations in Cross-Country Skiing
Source: Front Physiol. 2020 Oct 26;11:581339. doi: 10.3389/fphys.2020.581339 (PMC7649780; doi:10.3389/fphys.2020.581339)
Supplement: Supplementary file 1 [file Table_1.DOCX]

| **SUPPLEMENTARY TABLE 1: Physiological and performance characteristics in males and females** | | | | | | | | |
| --- | --- | --- | --- | --- | --- | --- | --- | --- |
| **Variable** | **PRE** | | **Effect size**  **Males vs females**  **PRE** | **POST1** | | **POST2** | | **Effect size**  **Males vs females**  **ΔPRE – POST2** |
| ***MALES (n = 17)*** |  |  |  |  |  |  |  |  |
| BW (kg) | 73.0 ± 9.0 | (12.3) | **1.06** | 72.1 ± 8.1 | (11.2) | 72.6 ± 7.5**^†^** | (10.3) | **0.89** |
| **TT_DP_** |  |  |  |  |  |  |  |  |
| seconds | 816.7 ± 57.0 | (7.0) | **2.28** | 811.6 ± 56.7 | (7.0) | 788.8 ± 54.7 | (6.9) | 0.13 |
| **RUN-VO_2max_** |  |  |  |  |  |  |  |  |
| mL · kg^-1^ · min^-1^ | 67.4 ± 6.7 | (9.9) | **1.91** | 69.6 ± 5.3 | (7.6) | 69.3 ± 7.1 | (10.2) | 0.46 |
| L · min^-1^ | 4.92 ± 0.68 | (13.8) | **2.41** | 5.01 ± 0.60 | (12.0) | 5.02 ± 0.62 | (12.4) | 0.04 |
| mL · kg^-0.67^ · min^-1^ | 277.4 ± 28.0 | (10.1) | **2.45** | 285.1 ± 22.6 | (7.9) | 284.6 ± 28.8 | (10.1) | 0.33 |
| HR | 196.9 ± 11.0 | (5.6) | 0.07 | 196.1 ± 11.6 | (5.9) | 195.2 ± 11.6 | (5.9) | 0.42 |
| RER | 1.13 ± 0.03 | (2.7) | 0.67 | 1.10 ± 0.05**^#^** | (4.5) | 1.14 ± 0.05****** | (4.4) | 0.20 |
| [La^-^_b_] | 9.7 ± 2.3 | (23.7) | 0.34 | 11.3 ± 2.5 | (22.1) | 10.3 ± 1.7**^†^** | (16.5) | **0.93** |
| RPE | 17.3 ± 1.4 | (8.1) | 0.17 | 17.8 ± 1.3 | (7.3) | 17.8 ± 1.4 | (7.9) | 0.05 |
| **DP-VO_2peak_** |  |  |  |  |  |  |  |  |
| mL · kg^-1^ · min^-1^ | 58.2 ± 6.2 | (10.7) | **1.72** | 58.5 ± 5.8 | (9.9) | 59.7 ± 5.6 | (9.4) | 0.21 |
| L · min^-1^ | 4.27 ± 0.63 | (14.8) | **2.23** | 4.24 ± 0.52 | (12.3) | 4.35 ± 0.56 | (12.9) | 0.20 |
| mL · kg^-0.67^ · min^-1^ | 239.9 ± 26.5 | (11.0) | **2.17** | 240.2 ± 23.2 | (9.7) | 245.5 ± 23.5 | (9.6) | 0.08 |
| %RUN-VO_2max_ | 86.7 ± 8.5 | (9.8) | 0.06 | 84.2 ± 6.1 | (7.2) | 86.4 ± 6.2 | (7.2) | 0.20 |
| HR | 191.8 ± 10.3 | (5.4) | 0.25 | 191.6 ± 10.4 | (5.4) | 191.4 ± 10.8 | (5.6) | 0.21 |
| RER | 1.10 ± 0.05 | (4.5) | 0.16 | 1.10 ± 0.05 | (4.5) | 1.13 ± 0.04 | (3.5) | 0.28 |
| [La^-^_b_] | 9.3 ± 2.3 | (24.7) | 0.10 | 8.7 ± 2.1 | (24.1) | 8.6 ± 1.8 | (20.9) | 0.63 |
| RPE | 17.4 ± 1.3 | (7.5) | 0.32 | 17.7 ± 1.2 | (6.8) | 17.3 ± 1.6 | (9.2) | 0.01 |
| TTE (s) | 567.9 ± 106.8 | (18.8) | **2.08** | 593.0 ± 106.3 | (17.9) | 613.2 ± 96.0 | (15.7) | 0.18 |
| **C_DP_ at LT** |  |  |  |  |  |  |  |  |
| mL · kg^-1^ · m^-1^ | 0.190 ± 0.019 | (10.0) | **0.97** | 0.191 ± 0.017**^#^** | (8.9) | 0.189 ± 0.019 | (10.1) | 0.81 |
| mL · kg^-0.67^ · m^-1^ | 0.782 ± 0.080 | (10.2) | **0.55** | 0.786 ± 0.073**^#^** | (9.3) | 0.778 ± 0.074 | (9.5) | 0.48 |
| **MAS** |  |  |  |  |  |  |  |  |
| m · min^-1^ | 309.4 ± 45.1 | (14.6) | **2.16** | 307.9 ± 39.5 | (12.8) | 317.0 ± 32.9 | (10.4) | 0.43 |
| km · h^-1^ | 18.6 ± 2.7 | (14.5) | **2.16** | 18.5 ± 2.4 | (13.0) | 19.0 ± 2.0 | (10.5) | 0.43 |
| **LT** |  |  |  |  |  |  |  |  |
| %DP-VO_2peak_ | 81.9 ± 5.5 | (6.7) | 0.13 | 84.0 ± 6.1**^#^** | (7.3) | 82.2 ± 5.9 | (7.2) | 0.37 |
| HR | 174.2 ± 11.6 | (6.7) | 0.27 | 172.9 ± 12.4 | (7.2) | 171.6 ± 12.9 | (7.5) | 0.19 |
| VO_2_ | 47.6 ± 5.3 | (11.1) | **1.24** | 48.9 ± 3.6 | (7.4) | 49.1 ± 5.8 | (11.8) | 0.48 |
| [La^-^_b_] | 4.7 ± 0.7 | (14.9) | 0.15 | 4.8 ± 0.7 | (14.6) | 4.6 ± 0.5 | (10.9) | 0.04 |
| Speed (km · h^-1^) | 15.1 ± 1.9 | (12.6) | **2.04** | 15.4 ± 1.4 | (9.1) | 15.6 ± 1.3 | (8.3) | 0.18 |
| **Strength** |  |  |  |  |  |  |  |  |
| 1RM half squat (kg) | 130.6 ± 20.3 | (15.5) | **1.22** | 140.3 ± 22.7 | (16.2) | 140.0 ± 21.4 | (15.3) | 0.06 |
| 1RM pull-down (kg) | 96.8 ± 12.5 | (12.9) | **1.86** | 96.2 ± 12.6 | (13.1) | 98.4 ± 12.6 | (12.8) | 0.41 |
| **Maximal power** |  |  |  |  |  |  |  |  |
| Half squat (w) | 905.1 ± 193.0 | (21.3) | **1.37** | 902.6 ± 165.8 | (18.4) | 923.8 ± 156.2 | (16.9) | 0.31 |
| Pull-down (w) | 544.8 ± 143.1 | (26.3) | **1.37** | 536.2 ± 102.7 | (19.2) | 560.2 ± 91.5 | (16.3) | 0.03 |
| SJ (cm) | 30.1 ± 5.5 | (18.3) | **1.21** | 28.9 ± 5.5 | (19.0) | 29.4 ± 5.1 | (17.3) | 0.11 |
| CMJ (cm) | 34.2 ± 5.6 | (16.4) | **1.52** | 33.7 ± 4.0 | (11.9) | 33.5 ± 4.7 | (14.0) | 0.12 |
| CMJas (cm) | 38.8 ± 5.1 | (13.1) | **1.75** | 37.1 ± 5.0 | (13.5) | 36.5 ± 4.8 | (13.2) | 0.21 |
|  |  |  |  |  |  |  |  |  |
| ***FEMALES (n = 12)*** |  |  |  |  |  |  |  |  |
| BW (kg) | 64.2 ± 7.4^§§^ | (11.5) | - | 64.5 ± 7.4^§^ | (11.4) | 65.4 ± 7.7^§^ | (11.8) | - |
| **TT_DP_** |  |  |  |  |  |  |  |  |
| seconds | 957.9 ± 66.7^§§^ | (7.0) | - | 945.2 ± 72.1^§§^ | (7.6) | 926.7 ± 57.1^§§^ | (6.2) | - |
| **RUN-VO_2max_** |  |  |  |  |  |  |  |  |
| mL · kg^-1^ · min^-1^ | 56.5 ± 4.5^§§^ | (8.0) | - | 57.8 ± 4.6^§§^ | (8.0) | 56.6 ± 4.3^§§^ | (7.6) | - |
| L · min^-1^ | 3.60 ± 0.37^§§^ | (10.3) | - | 3.72 ± 0.48^§§^ | (12.9) | 3.69 ± 0.47^§§^ | (12.7) | - |
| mL · kg^-0.67^ · min^-1^ | 222.3 ± 15.2^§§^ | (6.8) | - | 228.0 ± 19.0^§§^ | (8.3) | 224.4 ± 17.5^§§^ | (7.8) | - |
| HR | 196.2 ± 10.5 | (5.3) | - | 194.6 ± 9.5 | (4.9) | 191.2 ± 9.8 | (5.1) | - |
| RER | 1.11 ± 0.03 | (2.7) | - | 1.13 ± 0.05 | (4.4) | 1.14 ± 0.04 | (3.5) | - |
| [La^-^_b_] | 10.6 ± 3.0 | (28.3) | - | 11.3 ± 2.8 | (24.8) | 9.5 ± 2.6 | (27.4) | - |
| RPE | 17.0 ± 2.0 | (11.8) | - | 18.0 ± 0.9 | (5.0) | 17.4 ± 1.4 | (8.0) | - |
| **DP-VO_2peak_** |  |  |  |  |  |  |  |  |
| mL · kg^-1^ · min^-1^ | 48.8 ± 4.6^§§^ | (9.4) | - | 49.0 ± 5.0^§§^ | (10.2) | 49.5 ± 4.8^§§^ | (9.7) | - |
| L · min^-1^ | 3.11 ± 0.38^§§^ | (12.2) | - | 3.18 ± 0.47^§§^ | (14.8) | 3.24 ± 0.40^§§^ | (12.3) | - |
| mL · kg^-0.67^ · min^-1^ | 191.8 ± 16.7^§§^ | (8.7) | - | 193.9 ± 20.3^§§^ | (10.5) | 196.4 ± 17.5^§§^ | (8.9) | - |
| %RUN-VO_2max_ | 86.3 ± 5.5 | (6.4) | - | 84.9 ± 5.6 | (6.6) | 87.5 ± 5.2 | (5.9) | - |
| HR | 189.3 ± 9.3 | (4.9) | - | 189.8 ± 9.6 | (5.1) | 190.0 ± 8.9 | (4.7) | - |
| RER | 1.09 ± 0.07 | (6.4) | - | 1.12 ± 0.03 | (2.7) | 1.13 ± 0.07 | (6.2) | - |
| [La^-^_b_] | 9.1 ± 1.5 | (16.5) | - | 9.3 ± 1.5 | (16.1) | 9.6 ± 1.7 | (17.7) | - |
| RPE | 17.8 ± 1.2 | (6.7) | - | 17.4 ± 1.0 | (5.7) | 17.7 ± 1.2 | (6.8) | - |
| TTE (s) | 390.1 ± 56.7^§§^ | (14.5) | - | 426.2 ± 86.1^§§^ | (20.2) | 443.3 ± 85.4^§§^ | (19.3) | - |
| **C_DP_ at LT** |  |  |  |  |  |  |  |  |
| mL · kg^-1^ · m^-1^ | 0.209 ± 0.020^§§^ | (9.6) | - | 0.195 ± 0.022 | (11.3) | 0.197 ± 0.020 | (10.2) | - |
| mL · kg^-0.67^ · m^-1^ | 0.823 ± 0.070^§§^ | (8.5) | - | 0.769 ± 0.066 | (8.6) | 0.781 ± 0.057 | (7.3) | - |
| **MAS** |  |  |  |  |  |  |  |  |
| m · min^-1^ | 233.8 ± 20.5^§§^ | (8.8) | - | 253.6 ± 32.0^§§^ | (12.6) | 252.4 ± 27.1^§§^ | (10.7) | - |
| km · h^-1^ | 14.0 ± 1.2^§§^ | (8.6) | - | 15.2 ± 1.9^§§^ | (12.5) | 15.1 ± 1.6^§§^ | (10.6) | - |
| **LT** |  |  |  |  |  |  |  |  |
| %DP-VO_2peak_ | 82.8 ± 8.1 | (9.8) | - | 80.1 ± 6.0 | (7.5) | 80.7 ± 5.1 | (6.3) | - |
| HR | 177.3 ± 11.6 | (6.5) | - | 173.8 ± 11.8 | (6.8) | 173.3 ± 10.8 | (6.2) | - |
| VO_2_ | 40.5 ± 6.1^§§^ | (15.1) | - | 39.3 ± 5.2^§§^ | (13.2) | 39.9 ± 3.9^§§^ | (9.8) | - |
| [La^-^_b_] | 4.6 ± 0.6 | (13.0) | - | 4.5 ± 0.7 | (15.6) | 4.5 ± 0.7 | (15.6) | - |
| Speed (km · h^-1^) | 11.6 ± 1.5^§§^ | (12.9) | - | 12.2 ± 1.6^§§^ | (13.1) | 12.2 ± 1.3^§§^ | (10.7) | - |
| **Strength** |  |  |  |  |  |  |  |  |
| 1RM half squat (kg) | 107.7 ± 17.0^§§^ | (15.8) | - | 114.6 ± 17.8^§§^ | (15.5) | 119.2 ± 21.0^§§^ | (17.6) | - |
| 1RM pull-down (kg) | 74.2 ± 11.8^§§^ | (15.9) | - | 76.3 ± 11.5^§§^ | (15.1) | 78.3 ± 13.2^§§^ | (16.9) | - |
| **Maximal power** |  |  |  |  |  |  |  |  |
| Half squat (w) | 671.9 ± 144.0^§§^ | (21.4) | - | 694.9 ± 112.0^§§^ | (16.1) | 709.1 ± 134.7^§§^ | (19.0) | - |
| Pull-down (w) | 373.4 ± 104.4^§§^ | (28.0) | - | 375.5 ± 64.8^§§^ | (17.3) | 391.3 ± 93.3^§§^ | (23.8) | - |
| SJ (cm) | 24.9 ± 2.6^§§^ | (10.4) | - | 23.8 ± 3.2^§§^ | (13.4) | 24.3 ± 2.0^§§^ | (8.2) | - |
| CMJ (cm) | 27.7 ± 2.3^§§^ | (8.3) | - | 28.6 ± 2.2^§§^ | (7.7) | 27.0 ± 2.2^§§^ | (8.1) | - |
| CMJas (cm) | 31.7 ± 2.6^§§^ | (8.2) | - | 32.0 ± 2.7^§§^ | (8.4) | 29.8 ± 2.4^§§^ | (8.1) | - |
| Values are mean and standard deviation with coefficient of variance in parenthesis. Effect size is Cohen’s D value with significance p < 0.05 marked in bold. BW, body-weight. Kg. kilograms. TT_DP_, doublepoling time trial. RUN-VO_2max_, maximal oxygen uptake in running. mL · kg^-1^ · min^-1^, milliliters per kilogram bodyweight per minute. L · min^-1^, liters per minute. mL · kg^-0.67^ · min^-1^, milliliters per kilogram raised to the power of -0.67 per minute. HR, heart rate. RER, respiratory exchange ratio. [La^-^_b_], blood lactate concentration. RPE, rate of perceived exertion. %RUN-VO_2max_, fractional utilization of RUN-VO_2max_ at DP-VO_2peak._ TTE, time to exhaustion. C_DP_, oxygen cost of double poling at lactate threshold. mL · kg^-1^ · m^-1^, milliliters per kilogram per meter. mL · kg^-0.67^ · m^-1^, milliliters per kilogram raised to the power of -0.67 per meter. MAS, maximal aerobic speed. LT, lactate threshold. VO_2_, oxygen uptake. Km, kilometers. H, hours. 1RM, one repetition maximum. W, watt. SJ, squat jump. CMJ, counter movement jump. CMJas, counter movement jump with armswing. Cm, centimeters.  ** p < 0.01 significantly different from post 1 value.  ^§^ p < 0.05 significantly different from male value.  ^§§^ p < 0.01 significantly different from male value.  ^#^ p < 0.05 significantly different from female delta value from pre to post1  ^†^ p < 0.05 significantly different from female delta value from pre to post2 | | | | | | | | |
