# Supplementary material for: No Change – No Gain; The Effect of Age, Sex, Selected Genes and Training on Physiological and Performance Adaptations in Cross-Country Skiing
Source: Front Physiol. 2020 Oct 26;11:581339. doi: 10.3389/fphys.2020.581339 (PMC7649780; doi:10.3389/fphys.2020.581339)
Supplement: Supplementary file 2 [file Table_2.DOCX]

| **SUPPLEMENTARY TABLE 2: Physiological and performance characteristics in age-groups.** | | | | | | | | |
| --- | --- | --- | --- | --- | --- | --- | --- | --- |
| **Variable** | **PRE** | | **Effect size**  **16-18 yr vs ≥19 yr**  **PRE** | **POST1** | | **POST2** | | **Effect size**  **16-18 yr vs ≥19 yr**  **ΔPRE – POST2** |
| ***16 – 18 years (n = 16)*** |  |  |  |  |  |  |  |  |
| BW (kg) | 64.4 ± 6.7 | (10.4) | **1.45** | 64.8 ± 6.7 | (10.3) | 65.6 ± 6.4**^†^** | (9.8) | **0.94** |
| **TT_DP_** |  |  |  |  |  |  |  |  |
| seconds | 914.5 ± 92.0 | (10.1) | **1.08** | 907.9 ± 92.6 | (10.2) | 886.7 ± 81.9 | (9.2) | 0.13 |
| **RUN-VO_2max_** |  |  |  |  |  |  |  |  |
| mL · kg^-1^ · min^-1^ | 61.1 ± 8.0 | (13.1) | 0.52 | 61.4 ± 7.5**^#^** | (12.2) | 61.0 ± 8.0 | (13.1) | 0.69 |
| L · min^-1^ | 3.94 ± 0.68 | (17.3) | **1.33** | 3.98 ± 0.68 | (17.1) | 4.00 ± 0.68 | (17.0) | 0.20 |
| mL · kg^-0.67^ · min^-1^ | 241.3 ± 33.4 | (13.8) | **0.90** | 242.9 ± 32.3 | (13.3) | 242.5 ± 33.7 | (13.9) | 0.55 |
| HR | 201.6 ± 9.3 | (4.6) | **1.17** | 200.6 ± 9.0 | (4.5) | 198.1 ± 10.5 | (5.3) | 0.17 |
| RER | 1.12 ± 0.03 | (2.7) | 0.00 | 1.11 ± 0.05 | (4.5) | 1.14 ± 0.03 | (2.6) | 0.20 |
| [La^-^_b_] | 10.7 ± 2.4 | (22.4) | 0.55 | 11.8 ± 2.4 | (20.3) | 9.8 ± 2.0***^†^** | (20.4) | **0.92** |
| RPE | 16.7 ± 2.0 | (12.0) | **0.63** | 17.6 ± 1.2 | (6.8) | 17.0 ± 1.4 | (8.2) | 0.23 |
| **DP-VO_2peak_** |  |  |  |  |  |  |  |  |
| mL · kg^-1^ · min^-1^ | 51.5 ± 7.1 | (13.8) | **0.94** | 51.7 ± 7.1 | (13.7) | 52.6 ± 6.8 | (12.9) | 0.08 |
| L · min^-1^ | 3.32 ± 0.58 | (17.5) | **1.73** | 3.37 ± 0.58 | (17.2) | 3.45 ± 0.54 | (15.7) | 0.31 |
| mL · kg^-0.67^ · min^-1^ | 203.5 ± 29.2 | (14.3) | **1.33** | 204.9 ± 29.2 | (14.3) | 209.0 ± 27.4 | (13.1) | 0.05 |
| %RUN-VO_2max_ | 84.5 ± 6.2 | (7.3) | 0.63 | 84.3 ± 6.2 | (7.4) | 86.4 ± 5.7 | (6.6) | 0.48 |
| HF | 194.3 ± 9.3 | (4.8) | **0.85** | 195.2 ± 8.7 | (4.5) | 195.1 ± 9.1 | (4.7) | 0.36 |
| RER | 1.09 ± 0.04 | (3.7) | 0.18 | 1.12 ± 0.05 | (4.5) | 1.12 ± 0.05 | (4.5) | 0.00 |
| [La^-^_b_] | 9.6 ± 1.9 | (19.8) | 0.41 | 9.1 ± 2.2 | (24.2) | 9.1 ± 1.8 | (19.8) | 0.35 |
| RPE | 17.5 ± 1.4 | (8.0) | 0.10 | 17.4 ± 1.2 | (6.9) | 17.0 ± 1.7 | (10.0) | 0.61 |
| TTE (s) | 430.2 ± 92.4 | (21.5) | **1.35** | 455.0 ± 99.8 | (21.9) | 472.1 ± 95.6 | (20.2) | 0.35 |
| **C_DP_ at LT** |  |  |  |  |  |  |  |  |
| mL · kg^-1^ · m^-1^ | 0.205 ± 0.020 | (9.8) | **0.75** | 0.195 ± 0.018 | (9.2) | 0.196 ± 0.021 | (10.7) | 0.33 |
| mL · kg^-0.67^ · m^-1^ | 0.807 ± 0.074 | (9.2) | 0.21 | 0.771 ± 0.055 | (7.1) | 0.778 ± 0.062 | (8.0) | 0.23 |
| **MAS** |  |  |  |  |  |  |  |  |
| m · min^-1^ | 253.6 ± 38.1 | (15.0) | **1.83** | 266.4 ± 38.2 | (14.3) | 269.9 ± 39.4 | (14.6) | 0.36 |
| km · h^-1^ | 15.2 ± 2.3 | (15.1) | **1.83** | 16.0 ± 2.3 | (14.4) | 16.2 ± 2.4 | (14.8) | 0.36 |
| **LT** |  |  |  |  |  |  |  |  |
| %DP-VO_2peak_ | 81.5 ± 7.2 | (8.8) | 0.28 | 82.1 ± 7.6 | (9.3) | 82.0 ± 6.6 | (8.0) | 0.45 |
| HF | 179.7 ± 11.8 | (6.6) | **0.90** | 180.0 ± 10.0 | (5.6) | 178.0 ± 11.0 | (6.2) | 0.47 |
| VO_2_ | 42.0 ± 6.6 | (15.7) | **1.03** | 42.4 ± 7.0 | (16.5) | 43.1 ± 6.5 | (15.1) | 0.27 |
| [La^-^_b_] | 4.8 ± 0.5 | (10.4) | 0.45 | 4.8 ± 0.7 | (14.6) | 4.7 ± 0.6 | (12.8) | 0.00 |
| Speed (km · h^-1^) | 12.4 ± 1.9 | (15.3) | **1.41** | 13.1 ± 2.1 | (16.0) | 13.3 ± 2.0**^†^** | (15.0) | **0.87** |
| **Strength** |  |  |  |  |  |  |  |  |
| 1RM half squat (kg) | 112.5 ± 16.8 | (14.9) | **0.87** | 120.3 ± 20.0 | (16.6) | 123.4 ± 22.9 | (18.6) | 0.04 |
| 1RM pull-down (kg) | 78.1 ± 11.4 | (14.6) | **1.57** | 78.8 ± 11.0 | (14.0) | 80.9 ± 12.4 | (15.3) | 0.10 |
| **Maximal power** |  |  |  |  |  |  |  |  |
| Half squat (w) | 698.7 ± 153.4 | (22.0) | **1.43** | 748.2 ± 154.2**^##^** | (20.6) | 751.5 ± 165.4**^†^** | (22.0) | **0.88** |
| Pull-down (w) | 399.5 ± 126.7 | (31.7) | **1.27** | 417.1 ± 94.3 | (22.6) | 433.0 ± 107.5 | (24.8) | 0.52 |
| SJ | 28.1 ± 6.4 | (22.8) | 0.06 | 26.7 ± 4.7 | (17.6) | 27.1 ± 5.4 | (19.9) | 0.09 |
| CMJ | 31.5 ± 6.7 | (21.3) | 0.00 | 30.8 ± 4.5 | (14.6) | 30.4 ± 5.9 | (19.4) | 0.20 |
| CMJas | 35.5 ± 6.6 | (18.6) | 0.15 | 34.3 ± 5.5 | (16.0) | 33.1 ± 5.7 | (17.2) | 0.20 |
|  |  |  |  |  |  |  |  |  |
| ***≥ 19 years (n = 13)*** |  |  |  |  |  |  |  |  |
| BW (kg) | 75.5 ± 8.5**^§§^** | (11.3) | - | 74.1 ± 8.2**^§§^** | (11.1) | 74.6 ± 7.7**^§§^** | (10.3) | - |
| **TT_DP_** |  |  |  |  |  |  |  |  |
| seconds | 826.6 ± 70.0**^§§^** | (8.5) | - | 816.5 ± 61.6**^§§^** | (7.5) | 795.6 ± 69.3**^§§^** | (8.7) | - |
| **RUN-VO_2max_** |  |  |  |  |  |  |  |  |
| mL · kg^-1^ · min^-1^ | 65.2 ± 7.6 | (11.7) | - | 68.8 ± 5.9**^§^** | (8.6) | 67.8 ± 8.5**^§^** | (12.5) | - |
| L · min^-1^ | 4.92 ± 0.79**^§^** | (16.1) | - | 5.09 ± 0.62**^§§^** | (12.9) | 5.04 ± 0.72**^§§^** | (14.3) | - |
| mL · kg^-0.67^ · min^-1^ | 271.2 ± 33.4**^§^** | (12.3) | - | 284.2 ± 24.6**^§§^** | (8.7) | 280.9 ± 34.7**^§§^** | (12.4) | - |
| HR | 190.8 ± 9.1**^§§^** | (4.8) | - | 189.2 ± 9.1**^§§^** | (4.8) | 188.7 ± 9.4**^§^** | (5.0) | - |
| RER | 1.12 ± 0.03 | (2.7) | - | 1.11 ± 0.05 | (4.5) | 1.14 ± 0.05 | (4.4) | - |
| [La^-^_b_] | 9.3 ± 2.7 | (29.0) | - | 10.7 ± 2.8 | (26.2) | 10.2 ± 2.3 | (27.4) | - |
| RPE | 17.7 ± 1.0**^§^** | (5.6) | - | 18.2 ± 1.0 | (5.5) | 18.4 ± 0.9**^§§^** | (4.9) | - |
| **DP-VO_2peak_** |  |  |  |  |  |  |  |  |
| mL · kg^-1^ · min^-1^ | 57.7 ± 6.1**^§^** | (10.6) | - | 58.1 ± 5.8**^§^** | (10.0) | 59.1 ± 6.4**^§^** | (10.8) | - |
| L · min^-1^ | 4.36 ± 0.62**^§§^** | (14.2) | - | 4.33 ± 0.50**^§§^** | (11.5) | 4.42 ± 0.60**^§§^** | (13.6) | - |
| mL · kg^-0.67^ · min^-1^ | 240.2 ± 26.0**^§§^** | (10.8) | - | 240.8 ± 22.6**^§§^** | (16.1) | 245.1 ± 26.8**^§§^** | (10.9) | - |
| %RUN-VO_2max_ | 89.0 ± 8.0 | (9.0) | - | 84.6 ± 5.5 | (6.5) | 87.5 ± 5.9 | (6.7) | - |
| HF | 186.5 ± 9.0**^§^** | (4.8) | - | 185.5 ± 8.8**^§§^** | (4.7) | 185.5 ± 8.4**^§§^** | (4.5) | - |
| RER | 1.10 ± 0.07 | (6.4) | - | 1.10 ± 0.04 | (3.6) | 1.13 ± 0.06 | (5.3) | - |
| [La^-^_b_] | 8.8 ± 2.0 | (22.7) | - | 8.9 ± 1.5 | (16.9) | 9.0 ± 1.8 | (20.0) | - |
| RPE | 17.6 ± 1.1 | (6.3) | - | 17.7 ± 1.0 | (5.6) | 18.0 ± 0.7**^§^** | (3.9) | - |
| TTE (s) | 573.2 ± 117.5**^§§^** | (20.5) | - | 608.8 ± 107.7**^§§^** | (17.7) | 630.0 ± 97.9**^§§^** | (15.5) | - |
| **C_DP_ at LT** |  |  |  |  |  |  |  |  |
| mL · kg^-1^ · m^-1^ | 0.190 ± 0.020**^§^** | (10.5) | - | 0.190 ± 0.020 | (10.5) | 0.188 ± 0.018 | (9.6) | - |
| mL · kg^-0.67^ · m^-1^ | 0.790 ± 0.084 | (10.6) | - | 0.788 ± 0.085 | (10.8) | 0.781 ± 0.073 | (9.3) | - |
| **MAS** |  |  |  |  |  |  |  |  |
| m · min^-1^ | 308.4 ± 23.8**^§§^** | (7.7) | - | 308.8 ± 43.0**^§§^** | (13.9) | 315.3 ± 37.3**^§§^** | (11.8) | - |
| km · h^-1^ | 18.5 ± 1.4**^§§^** | (7.6) | - | 18.5 ± 2.6**^§§^** | (14.1) | 18.9 ± 2.2**^§§^** | (11.6) | - |
| **LT** |  |  |  |  |  |  |  |  |
| %DP-VO_2peak_ | 83.3 ± 5.8 | (7.0) | - | 82.3 ± 4.4 | (5.3) | 81.0 ± 4.2 | (5.2) | - |
| HF | 170.2 ± 9.1**^§^** | (5.3) | - | 164.9 ± 8.5**^§§^** | (5.2) | 165.2 ± 9.0**^§§^** | (5.4) | - |
| VO_2_ | 48.0 ± 4.9**^§^** | (10.2) | - | 48.0 ± 4.2**^§^** | (8.8) | 47.9 ± 6.5**^§^** | (13.6) | - |
| [La^-^_b_] | 4.5 ± 0.8 | (17.8) | - | 4.6 ± 0.7 | (15.2) | 4.3 ± 0.5 | (11.6) | - |
| Speed (km · h^-1^) | 15.3 ± 2.2**^§§^** | (14.4) | - | 15.3 ± 1.7**^§§^** | (11.1) | 15.3 ± 1.7**^§§^** | (11.1) | - |
| **Strength** |  |  |  |  |  |  |  |  |
| 1RM half squat (kg) | 130.4 ± 23.8**^§^** | (18.3) | - | 141.2 ± 24.6**^§^** | (17.4) | 141.3 ± 20.5**^§^** | (14.5) | - |
| 1RM pull-down (kg) | 98.8 ± 14.7**^§§^** | (14.9) | - | 99.2 ± 12.7**^§§^** | (12.8) | 101.7 ± 12.9**^§§^** | (12.7) | - |
| **Maximal power** |  |  |  |  |  |  |  |  |
| Half squat (w) | 943.9 ± 187.6**^§§^** | (19.9) | - | 900.9 ± 172.6**^§^** | (19.2) | 938.8 ± 144.7**^§§^** | (15.4) | - |
| Pull-down (w) | 565.4 ± 134.0**^§§^** | (23.7) | - | 534.4 ± 117.2**^§§^** | (21.9) | 560.8 ± 107.9**^§§^** | (19.2) | - |
| SJ | 27.8 ± 3.2 | (11.5) | - | 26.9 ± 4.8 | (17.8) | 27.4 ± 4.1 | (15.0) | - |
| CMJ | 31.5 ± 3.8 | (12.1) | - | 32.6 ± 3.7 | (11.3) | 31.2 ± 3.7 | (11.9) | - |
| CMJas | 36.3 ± 3.9 | (10.7) | - | 35.8 ± 4.0 | (11.2) | 34.4 ± 4.4 | (12.8) | - |
| Values are mean and standard deviation with coefficient of variance in percentage in parenthesis. Effect size is Cohen’s D value with significance p < 0.05 marked in bold. BW, body-weight. Kg. kilograms. TT_DP_, double poling time trial. RUN-VO_2max_, maximal oxygen uptake in running. mL · kg^-1^ · min^-1^, milliliters per kilogram bodyweight per minute. L · min^-1^, liters per minute. mL · kg^-0.67^ · min^-1^, milliliters per kilogram raised to the power of -0.67 per minute. HR, heart rate. RER, respiratory exchange ratio. [La^-^_b_], blood lactate concentration. RPE, rate of perceived exertion. %RUN-VO_2max_, fractional utilization of RUN-VO_2max_ at DP-VO_2peak._ TTE, time to exhaustion. C_DP_, oxygen cost of double poling at lactate threshold. mL · kg^-1^ · m^-1^, milliliters per kilogram per meter. mL · kg^-0.67^ · m^-1^, milliliters per kilogram raised to the power of -0.67 per meter. MAS, maximal aerobic speed. LT, lactate threshold. VO_2_, oxygen uptake. Km, kilometers. H, hours. 1RM, one repetition maximum. W, watt. SJ, squat jump. CMJ, counter movement jump. CMJas, counter movement jump with armswing. Cm, centimeters.  ** p < 0.01 significantly different from post 1 value.  ^#^ p < 0.05 significantly different from ≥ 19 years delta value from pre to post1.  ^##^ p < 0.01 significantly different from ≥ 19 years delta value from pre to post1.  **^†^** p<0.05 significantly different from ≥ 19 years delta value from pre to post2.  ^§^ p < 0.05 significantly different from 16 – 18 years value.  ^§§^ p < 0.01 significantly different from 16 – 18 years value. | | | | | | | | |
