# Supplementary material for: No Change – No Gain; The Effect of Age, Sex, Selected Genes and Training on Physiological and Performance Adaptations in Cross-Country Skiing
Source: Front Physiol. 2020 Oct 26;11:581339. doi: 10.3389/fphys.2020.581339 (PMC7649780; doi:10.3389/fphys.2020.581339)
Supplement: Supplementary file 3 [file Table_3.DOCX]

| **SUPPLEMENTARY TABLE 3: All training characteristics during the 6 months study period (n = 29)** | | | | | | | |
| --- | --- | --- | --- | --- | --- | --- | --- |
| **Variable** | **1. training period (May – July)** | | |  | **2. training period (August – October)** | | |
|  | ***P*_1A_** | ***P*_1B_** | ***P*_1_** |  | ***P*_2A_** | ***P*_2B_** | ***P*_2_** |
| **Duration (weeks)** | 6.3 ± 0.8 | 6.4 ± 0.9 | 12.7 ± 1.7 |  | 5.3 ± 0.7 | 5.4 ± 0.7 | 10.7 ± 1.4 |
| **Training (min · week^-1^)** |  |  |  |  |  |  |  |
| Mean total training volume | 677.4 ± 165.9 | 724.0 ± 201.8 | 701.5 ± 169.8 |  | 782.0 ± 151.2 | 712.6 ± 171.9 | 753.2 ± 137.6* |
| Endurance training |  |  |  |  |  |  |  |
| LIT | 521.9 ± 144.7 | 574.4 ± 176.9 | 548.7 ± 148.2 |  | 582.5 ± 160.8 | 537.6 ± 152.6 | 569.1 ± 116.9 |
| MIT | 27.8 ± 13.6 | 30.8 ± 14.8 | 29.4 ± 11.4 |  | 30.3 ± 15.9 | 29.8 ± 18.0 | 30.4 ± 14.7 |
| HIT | 34.0 ± 15.6 | 29.4 ± 18.5 | 31.8 ± 15.7 |  | 38.9 ± 21.7 | 32.5 ± 15.5 | 36.0 ± 17.2 |
| Total | 583.7 ± 145.3 | 634.5 ± 190.6 | 609.8 ± 154.1 |  | 662.0 ± 137.1 | 599.9 ± 164.9 | 635.5 ± 126.3 |
| Training mode |  |  |  |  |  |  |  |
| Ski specific | 276.9 ± 116.4 | 322.1 ± 145.6 | 303.1 ± 120.1 |  | 377.3 ± 128.3^§^ | 333.5 ± 126.0 | 353.8 ± 105.4** |
| LIT_ski_ | 251.1 ± 107.1 | 287.5 ± 128.9 | 270.2 ± 108.0 |  | 334.8 ± 111.9^§^ | 301.2 ± 114.1 | 313.6 ± 91.0** |
| MIT_ski_ | 14.5 ± 12.5 | 18.1 ± 11.3 | 15.7 ± 8.4 |  | 19.7 ± 12.0 | 19.6 ± 16.5 | 19.4 ± 12.4 |
| HIT_ski_ | 11.4 ± 8.5 | 16.5 ± 16.4 | 14.0 ± 11.7 |  | 22.8 ± 15.0^¤§§^ | 12.6 ± 8.4 | 17.5 ± 10.6 |
| Running | 247.1 ± 80.0 | 236.7 ± 96.1 | 244.5 ± 77.6 |  | 245.8 ± 74.3 | 237.0 ± 109.4 | 245.4 ± 71.8 |
| Cycling | 52.3 ± 104.5 | 67.7 ± 94.6 | 60.5 ± 95.5 |  | 47.1 ± 63.2 | 26.1 ± 37.1 | 35.3 ± 46.2 |
|  |  |  |  |  |  |  |  |
| Strength training | 67.5 ± 38.3 | 55.6 ± 30.7 | 61.7 ± 30.5 |  | 87.5 ± 35.7^††^ | 67.2 ± 34.6 | 77.8 ± 31.4** |
| Speed/jump training | 7.7 ± 7.5 | 8.7 ± 10.6 | 8.2 ± 8.4 |  | 11.0 ± 11.1 | 7.9 ± 10.2 | 9.6 ± 10.4 |
| Other | 18.4 ± 41.7 | 24.9 ± 45.2 | 21.7 ± 41.8 |  | 21.4 ± 31.5 | 37.6 ± 39.7 | 30.5 ± 33.9 |
| Values are mean and standard deviation. min · week^-1^, minutes per week. *P*_1_, first training period from May to October. *P*_1A_, first half of the first preparation period. *P*_1B_, second half of the first preparation period. *P*_2A_, first half of the second preparation period. *P*_2B_, second half of the second preparation period. LIT, low-intensity training. MIT, moderate-intensity training, HIT, high-intensity training.  * p < 0.05 significantly different from *P*_1_ value.  ** p < 0.01 significantly different from *P*_1_ value.  ^§^ p < 0.05 significantly different from *P*_1A_ value.  ^§§^ p < 0.01 significantly different from *P*_1A_ value.  ^¤^ p < 0.05 significantly different from *P*_2B_ value.  ^††^ p < 0.01 significantly different from *P*_1B_ value. | | | | | | | |
