# Supplementary material for: No Change – No Gain; The Effect of Age, Sex, Selected Genes and Training on Physiological and Performance Adaptations in Cross-Country Skiing
Source: Front Physiol. 2020 Oct 26;11:581339. doi: 10.3389/fphys.2020.581339 (PMC7649780; doi:10.3389/fphys.2020.581339)
Supplement: Supplementary file 4 [file Table_4.DOCX]

| **SUPPLEMENTARY TABLE 3: Training characteristics during the 6 months study period in males and females.** | | | | | | | |
| --- | --- | --- | --- | --- | --- | --- | --- |
| **Variable** | **1. training period (May – July)** | | |  | **2. training period (August – October)** | | |
|  | ***P*_1A_** | ***P*_1B_** | ***P*_1_** |  | ***P*_2A_** | ***P*_2B_** | ***P*_2_** |
| ***MALES (n = 17)*** |  |  |  |  |  |  |  |
| **Duration (weeks)** | 6.5 ± 0.9 | 6.6 ± 0.9 | 13.2 ± 1.8 |  | 5.2 ± 0.5 | 5.3 ± 0.5 | 10.6 ± 1.2 |
| **Training (min · week^-1^)** |  |  |  |  |  |  |  |
| Mean total training volume | 677.5 ± 199.9 | 758.2 ± 221.4 | 717.5 ± 200.9 |  | 809.3 ± 187.7 | 720.7 ± 204.9 | 769.1 ± 171.3 |
| Endurance training |  |  |  |  |  |  |  |
| LIT | 518.1 ± 167.1 | 605.7 ± 188.8 | 561.2 ± 168.7 |  | 599.7 ± 200.8 | 559.1 ± 184.1 | 591.3 ± 142.8 |
| MIT | 27.7 ± 15.2 | 34.9 ± 16.5 | 31.4 ± 13.3 |  | 34.3 ± 18.8 | 33.7 ± 21.1 | 34.2 ± 17.3 |
| HIT | 37.9 ± 13.3 | 32.6 ± 17.3 | 35.2 ± 13.5 |  | 46.2 ± 20.5**^††^** | 34.7 ± 14.8 | 40.7 ± 16.2 |
| Total | 583.7 ± 170.3 | 673.2 ± 198.1 | 627.8 ± 173.2 |  | 697.9 ± 157.3 | 627.5 ± 198.0 | 666.2 ± 150.9 |
| Training mode |  |  |  |  |  |  |  |
| Ski specific | 269.2 ± 131.4 | 324.2 ± 170.4 | 297.7 ± 139.9 |  | 385.9 ± 159.9 | 335.8 ± 147.0 | 362.4 ± 126.7****** |
| LIT_ski_ | 243.7 ± 119.1 | 290.4 ± 153.3 | 267.6 ± 126.8 |  | 341.0 ± 140.3 | 303.2 ± 130.3 | 321.4 ± 112.9****** |
| MIT_ski_ | 14.1 ± 15.1 | 17.7 ± 12.0 | 14.8 ± 9.0 |  | 20.6 ± 15.1 | 20.7 ± 19.7 | 20.5 ± 15.3***** |
| HIT_ski_ | 11.3 ± 7.0 | 16.1 ± 15.1 | 13.8 ± 9.8 |  | 24.3 ± 15.5 | 11.9 ± 8.0 | 17.9 ± 10.3 |
| Running | 226.4 ± 85.7 | 249.4 ± 108.4 | 240.8 ± 88.7 |  | 256.4 ± 83.7 | 244.0 ± 138.3 | 252.6 ± 87.3 |
| Cycling | 77.0 ± 130.4**^†^** | 85.5 ± 111.7 | 81.9 ± 116.6 |  | 69.9 ± 74.1 | 35.9 ± 44.8 | 50.7 ± 55.1**^†^** |
|  |  |  |  |  |  |  |  |
| Strength training | 67.6 ± 46.3 | 52.8 ± 32.9 | 60.3 ± 35.6 |  | 85.9 ± 38.5 | 60.5 ± 33.5 | 73.5 ± 33.0***** |
| Speed/jump training | 3.9 ± 3.4**^††^** | 3.2 ± 4.2**^††^** | 3.5 ± 3.0**^††^** |  | 6.1 ± 4.7**^††^** | 3.1 ± 3.3**^††^** | 4.7 ± 3.7**^†^** |
| Other | 22.4 ± 53.7 | 29.0 ± 54.5 | 25.8 ± 52.8 |  | 19.4 ± 30.9 | 29.6 ± 44.7 | 25.0 ± 38.1 |
|  |  |  |  |  |  |  |  |
| ***FEMALES (n = 12)*** |  |  |  |  |  |  |  |
| **Duration (weeks)** | 6.0 ± 0.7 | 6.0 ± 0.7 | 12.0 ± 1.4 |  | 5.6 ± 0.9 | 5.6 ± 0.8 | 10.9 ± 1.7 |
| **Training (min · week^-1^)** |  |  |  |  |  |  |  |
| Mean total training volume | 677.2 ± 111.5 | 675.5 ± 167.4 | 678.8 ± 117.2 |  | 743.2 ± 64.5 | 701.1 ± 118.0 | 730.8 ± 67.6 |
| Endurance training |  |  |  |  |  |  |  |
| LIT | 527.2 ± 112.3 | 529.9 ± 155.2 | 530.9 ± 118.1 |  | 558.0 ± 77.6 | 507.0 ± 90.8 | 537.7 ± 57.4 |
| MIT | 28.0 ± 11.7 | 25.0 ± 9.9 | 26.6 ± 8.0 |  | 24.7 ± 8.3 | 24.3 ± 11.2 | 25.0 ± 7.7 |
| HIT | 28.5 ± 17.6 | 24.9 ± 19.9 | 26.8 ± 17.9 |  | 28.5 ± 19.7 | 29.5 ± 16.5 | 29.4 ± 16.9 |
| Total | 583.7 ± 107.4 | 579.8 ± 172.7 | 584.3 ± 124.9 |  | 611.2 ± 83.8 | 560.8 ± 96.7 | 592.1 ± 62.9 |
| Training mode |  |  |  |  |  |  |  |
| Ski specific | 287.8 ± 95.5 | 319.2 ± 108.1 | 310.8 ± 90.3 |  | 365.2 ± 66.6 | 330.2 ± 94.7 | 341.8 ± 57.1 |
| LIT_ski_ | 261.4 ± 91.4 | 283.4 ± 89.9 | 273.9 ± 79.2 |  | 326.0 ± 55.5 | 298.5 ± 91.9 | 302.6 ± 47.8 |
| MIT_ski_ | 14.9 ± 8.1 | 18.7 ± 10.8 | 16.9 ± 7.8 |  | 18.4 ± 5.9 | 18.1 ± 11.1 | 17.8 ± 6.8 |
| HIT_ski_ | 11.5 ± 10.6 | 17.1 ± 18.7 | 14.4 ± 14.5 |  | 20.7 ± 14.7 | 13.7 ± 9.1 | 16.9 ± 11.5 |
| Running | 276.4 ± 63.4 | 218.6 ± 76.3 | 249.8 ± 61.9 |  | 230.6 ± 58.7 | 227.0 ± 49.4 | 235.2 ± 43.1 |
| Cycling | 17.4 ± 28.2 | 42.4 ± 58.5 | 30.1 ± 41.4 |  | 14.8 ± 15.0 | 12.2 ± 14.6 | 13.5 ± 11.5 |
|  |  |  |  |  |  |  |  |
| Strength training | 67.4 ± 24.9 | 59.7 ± 28.1 | 63.5 ± 22.7 |  | 89.8 ± 33.0 | 76.7 ± 35.3 | 83.8 ± 29.3****** |
| Speed/jump training | 13.3 ± 8.3 | 16.6 ± 12.0 | 14.9 ± 9.0 |  | 17.8 ± 14.0 | 14.7 ± 12.8 | 16.5 ± 13.0 |
| Other | 12.9 ± 13.3 | 19.0 ± 28.2 | 16.0 ± 18.2 |  | 24.4 ± 33.6 | 48.8 ± 29.4**^§^** | 38.4 ± 26.5***^#^** |
| Values are mean and standard deviation. min · week^-1^, minutes per week. *P*_1_, first training period from May to July. *P*_1A_, first half of the first training period. *P*_1B_, second half of the first training period. *P*_2_, second training period from August to October. *P*_2A_, first half of the second training period. *P*_2B_, second half of the second training period. LIT, low-intensity training. MIT, moderate-intensity training, HIT, high-intensity training.  * p < 0.05 significantly different from *P*_1_ value.  ** p < 0.01 significantly different from *P*_1_ value.  ^#^ p < 0.05 significantly different in delta values in other training from male value.  **^†^** p < 0.05 significantly different from female value.  **^††^** p < 0.01 significantly different from female value.  ^§^ p < 0.05 significantly different from *P*_1A_ and *P*_1B_. | | | | | | | |
