# Supplementary material for: No Change – No Gain; The Effect of Age, Sex, Selected Genes and Training on Physiological and Performance Adaptations in Cross-Country Skiing
Source: Front Physiol. 2020 Oct 26;11:581339. doi: 10.3389/fphys.2020.581339 (PMC7649780; doi:10.3389/fphys.2020.581339)
Supplement: Supplementary file 5 [file Table_5.DOCX]

| **SUPPLEMENTARY TABLE 5: Training characteristics during the 6 months study period in 16 – 18 years and ≥19 years.** | | | | | | | |
| --- | --- | --- | --- | --- | --- | --- | --- |
| **Variable** | **1. preparation period (May – July)** | | |  | **2. preparation period (August – October)** | | |
|  | ***P*_1A_** | ***P*_1B_** | ***P*_1_** |  | ***P*_2A_** | ***P*_2B_** | ***P*_2_** |
| ***16 – 18 YEARS (n = 16)*** |  |  |  |  |  |  |  |
| **Duration (weeks)** | 6.3 ± 0.9 | 6.3 ± 0.9 | 12.6 ± 1.7 |  | 5.2 ± 0.8 | 5.3 ± 0.8 | 10.4 ± 1.1 |
| **Training (min · week^-1^)** |  |  |  |  |  |  |  |
| Mean total training volume | 671.7 ± 154.6 | 689.6 ± 205.4 | 681.1 ± 163.4 |  | 802.4 ± 162.2 | 687.9 ± 162.9 | 754.5 ± 135.3****** |
| Endurance training |  |  |  |  |  |  |  |
| LIT | 499.1 ± 128.4 | 520.8 ± 168.2**^#^** | 510.1 ± 133.3 |  | 578.5 ± 179.2 | 481.2 ± 130.7**^#^** | 544.7 ± 102.9 |
| MIT | 24.4 ± 10.9 | 26.0 ± 9.5 | 25.3 ± 7.2 |  | 23.4 ± 13.1**^##^** | 24.6 ± 11.9**^#^** | 24.6 ± 10.1 |
| HIT | 31.9 ± 15.2 | 28.4 ± 17.5 | 30.2 ± 14.8 |  | 34.3 ± 20.5 | 32.0 ± 17.1 | 33.7 ± 17.1 |
| Total | 555.4 ± 130.7 | 575.2 ± 182.8 | 565.6 ± 140.8 |  | 655.0 ± 142.3 | 537.8 ± 137.4 | 602.9 ± 112.8 |
| Training mode |  |  |  |  |  |  |  |
| Ski specific | 251.5 ± 92.7**^§§^** | 287.8 ± 128.1**^§^** | 273.6 ± 99.4 |  | 389.2 ± 121.3 | 279.6 ± 65.4**^#§^** | 339.0 ± 82.2****** |
| LIT_ski_ | 228.1 ± 87.7**^§§^** | 256.3 ± 114.1**^§^** | 242.6 ± 90.4 |  | 348.7 ± 104.7 | 251.5 ± 64.3**^§^** | 300.3 ± 73.6****** |
| MIT_ski_ | 12.6 ± 7.5 | 15.9 ± 10.9 | 14.3 ± 8.0 |  | 17.9 ± 12.4 | 16.7 ± 11.0 | 17.3 ± 9.6 |
| HIT_ski_ | 10.8 ± 5.9 | 15.6 ± 13.1 | 13.3 ± 8.5 |  | 22.5 ± 14.3 | 11.5 ± 8.1 | 17.0 ± 9.4 |
| Running | 242.3 ± 73.8 | 223.7 ± 79.3 | 236.0 ± 65.6 |  | 246.7 ± 77.6 | 208.4 ± 95.9 | 232.9 ± 57.2 |
| Cycling | 54.2 ± 104.7 | 61.6 ± 90.3 | 58.3 ± 92.6 |  | 34.5 ± 48.7 | 27.8 ± 41.0 | 29.1 ± 42.3 |
|  |  |  |  |  |  |  |  |
| Strength training | 79.0 ± 33.0**^#^** | 61.7 ± 31.9**^§§^** | 70.4 ± 26.2 |  | 105.2 ± 26.7**^##^** | 77.9 ± 37.2 | 92.3 ± 26.9****^##^** |
| Speed/jump training | 8.4 ± 7.5 | 12.0 ± 11.7**^#^** | 10.2 ± 9.1 |  | 15.2 ± 13.0**^##^** | 11.9 ± 12.1**^#^** | 13.7 ± 12.2**^#^** |
| Other | 28.9 ± 54.0 | 40.4 ± 56.5**^#^** | 34.8 ± 53.1**^#^** |  | 27.1 ± 30.3 | 60.2 ± 36.9**^##^** | 45.5 ± 32.3**^##^** |
|  |  |  |  |  |  |  |  |
| ***≥ 19 YEARS (n = 13)*** |  |  |  |  |  |  |  |
| **Duration (weeks)** | 6.3 ± 0.8 | 6.4 ± 0.9 | 12.8 ± 1.7 |  | 5.3 ± 0.7 | 5.4 ± 0.7 | 11.1 ± 1.6 |
| **Training (min · week^-1^)** |  |  |  |  |  |  |  |
| Mean total training volume | 684.4 ± 185.0 | 766.2 ± 196.9 | 726.6 ± 180.7 |  | 756.9 ± 130.6 | 743.0 ± 184.3 | 751.7 ± 145.9 |
| Endurance training |  |  |  |  |  |  |  |
| LIT | 549.9 ± 163.3 | 640.3 ± 170.5 | 596.1 ± 157.0 |  | 587.3 ± 141.8 | 607.0 ± 153.3 | 599.2 ± 129.9 |
| MIT | 32.0 ± 15.8 | 36.6 ± 18.1 | 34.4 ± 13.9 |  | 38.8 ± 15.2 | 36.1 ± 22.4 | 37.5 ± 16.6 |
| HIT | 36.6 ± 16.4 | 30.7 ± 20.2 | 33.7 ± 17.2 |  | 44.4 ± 22.7 | 33.2 ± 13.9 | 38.9 ± 17.5 |
| Total | 583.7 ± 145.3 | 634.5 ± 190.6 | 664.2 ± 157.6 |  | 662.0 ± 137.1 | 599.9 ± 164.9 | 675.6 ± 134.7 |
| Training mode |  |  |  |  |  |  |  |
| Ski specific | 308.1 ± 137.6 | 364.4 ± 159.5 | 339.5 ± 136.8 |  | 362.6 ± 140.1 | 399.7 ± 151.9 | 372.0 ± 129.7 |
| LIT_ski_ | 279.3 ± 124.8 | 325.9 ± 139.9 | 304.2 ± 121.4 |  | 317.7 ± 122.2 | 362.5 ± 133.6 | 330.0 ± 109.6 |
| MIT_ski_ | 16.7 ± 16.9 | 20.8 ± 11.7 | 17.4 ± 9.0 |  | 21.8 ± 11.8 | 23.3 ± 21.4 | 21.9 ± 15.2 |
| HIT_ski_ | 12.1 ± 11.2 | 17.7 ± 20.2 | 15.0 ± 15.1 |  | 23.2 ± 16.2 | 13.9 ± 8.8 | 18.1 ± 12.3 |
| Running | 253.0 ± 89.8 | 252.6 ± 114.9 | 254.9 ± 91.9 |  | 244.6 ± 73.3 | 272.2 ± 118.3 | 260.8 ± 86.5 |
| Cycling | 52.3 ± 104.5 | 67.7 ± 94.6 | 63.1 ± 102.7 |  | 47.1 ± 63.2 | 26.1 ± 37.1 | 42.9 ± 51.3 |
|  |  |  |  |  |  |  |  |
| Strength training | 67.5 ± 38.3 | 55.6 ± 30.7 | 50.9 ± 32.8 |  | 87.5 ± 35.7 | 67.2 ± 34.6 | 60.0 ± 27.9***** |
| Speed/jump training | 7.7 ± 7.5 | 8.7 ± 10.6 | 5.8 ± 6.9 |  | 11.0 ± 11.1 | 7.9 ± 10.2 | 4.5 ± 4.0 |
| Other | 18.4 ± 41.7 | 24.9 ± 45.2 | 5.7 ± 6.9 |  | 21.4 ± 31.5 | 37.6 ± 39.7 | 12.0 ± 26.7 |
| Values are mean and standard deviation. min · week^-1^, minutes per week. *P*_1_, first training period from May to July. *P*_1A_, first half of the first training period. *P*_1B_, second half of the first training period. *P*_2_, second training period from August to October. *P*_2A_, first half of the second training period. *P*_2B_, second half of the second training period. LIT, low-intensity training. MIT, moderate-intensity training, HIT, high-intensity training.  *p < 0.05 significantly different within age-groups from *P*_1_ value.  ** p < 0.01 significantly different within age-groups from *P*_1_ value.  **^#^** p < 0.05 significantly different from ≥ 19 yr value.  **^##^** p < 0.01 significantly different from ≥ 19 yr value.  ^§^ p < 0.05 significantly different from *P*_2A_ value.  ^§§^ p < 0.01 significantly different from *P*_2A_ value. | | | | | | | |
