# Supplementary material for: No Change – No Gain; The Effect of Age, Sex, Selected Genes and Training on Physiological and Performance Adaptations in Cross-Country Skiing
Source: Front Physiol. 2020 Oct 26;11:581339. doi: 10.3389/fphys.2020.581339 (PMC7649780; doi:10.3389/fphys.2020.581339)
Supplement: Supplementary file 7 [file Table_7.docx]

| Supplementary table: Physiological baseline results divided by *ACTN3* and *ACE* genotypes (N=40) | | | | | | |
| --- | --- | --- | --- | --- | --- | --- |
|  | *ACTN3* | | | *ACE* | | |
| Genotype (N) | **RR (12)** | **RX (18)** | **XX (10)** | **DD (12)** | **ID (21)** | **II (7)** |
| Independent of gender | | | | | | |
| RUN-VO_2max_ | 250.0 ± 46.6 | 270.0 ± 35.6 | 267.8 ± 27.0 | 263.7 ± 49.3 | 261.5 ± 32.1 | 268.7 ± 35.5 |
| DP-VO_2peak_ | 212.4 ± 36.8 | 234.1 ± 35.5 | 227.6 ± 22.0 | 229.3 ± 44.7 | 223.8 ± 27.3 | 226.9 ± 34.1 |
| C_DP_ | 0.798 ± 0.067 | 0.780 ± 0.101 | 0.830 ± 0.071 | 0.762 ± 0.090 | 0.814 ± 0.071 | 0.809 ± 0.112 |
| 1RM half squat | 118.1 ± 26.0 | 125.8 ± 26.4 | 133.5 ± 28.2 | 131.0 ± 30.9 | 121.7 ± 21.4 | 127.1 ± 34.9 |
| 1RM pull-down | 91.2 ± 19.2 | 87.8 ± 19.3 | 93.5 ± 17.3 | 95.8 ± 15.8 | 89.8 ± 18.8 | 82.9 ± 22.5 |
|  |  |  |  |  |  |  |
| Corrected for gender | | | | | | |
| RUN-VO_2max_ | 273.1 ± 25.7 | 290.0 ± 25.3 | 275.1 ± 21.6 | 278.7 ± 27.5 | 278.9 ± 26.5 | 292.6 ± 15.5 |
| DP-VO_2peak_ | 232.4 ± 20.0 | 253.7 ± 28.8* | 237.8 ± 11.9 | 248.0 ± 26.3 | 239.4 ± 24.8 | 247.2 ± 22.0 |
| C_DP_ | 0.781 ± 0.065 | 0.770 ± 0.097 | 0.816 ± 0.067 | 0.755 ± 0.095 | 0.800 ± 0.067 | 0.790 ± 0.098 |
| 1RM half squat | 126.6 ± 20.7 | 133.0 ± 22.7 | 133.9 ± 23.5 | 132.9 ± 21.6 | 128.8 ± 20.4 | 136.1 ± 29.1 |
| 1RM pull-down | 101.2 ± 14.0 | 96.6 ± 16.6 | 98.2 ± 11.9 | 104.3± 9.1 | 97.4 ± 16.1 | 91.3 ± 15.4 |
| Values are mean ± standard deviation. RUN-VO_2max_, maximal oxygen uptake in running expressed in millilitres per kilogram bodyweight raised to the power of -0.67 per minute. DP-VO_2peak_, peak oxygen uptake in double-poling expressed in millilitres per kilogram bodyweight raised to the power of -0.67 per minute. C_DP_, oxygen cost of double-poling expressed in millilitres per kilogram bodyweight raised to the power of -0.67 per meter. 1RM, one repetition maximum expressed in kilograms.  * p < 0.05 significantly different from *ACTN3* RR genotype | | | | | | |
